# The neuropathology of bipolar disorder: systematic review and meta-analysis

**DOI:** 10.1038/s41380-018-0213-3

**Published:** 2018-08-20

**Authors:** Paul J. Harrison, Lucy Colbourne, Charlotte H. Harrison

**Affiliations:** 1 Department of Psychiatry, University of Oxford, Warneford Hospital, Oxford, OX3 7JX UK; 2 Oxford Health NHS Foundation Trust, Warneford Hospital, Oxford, UK; 3 Faculty of Medicine, University of Southampton, Southampton General Hospital, Southampton, UK

**Keywords:** Bipolar disorder, Neuroscience

## Abstract

Various neuropathological findings have been reported in bipolar disorder (BD). However, it is unclear which findings are well established. To address this gap, we carried out a systematic review of the literature. We searched over 5000 publications, identifying 103 data papers, of which 81 were eligible for inclusion. Our main findings can be summarised as follows. First, most studies have relied on a limited number of brain collections, and have used relatively small sample sizes (averaging 12 BD cases and 15 controls). Second, surprisingly few studies have attempted to replicate closely a previous one, precluding substantial meta-analyses, such that the latter were all limited to two studies each, and comprising 16–36 BD cases and 16–74 controls. As such, no neuropathological findings can be considered to have been established beyond reasonable doubt. Nevertheless, there are several replicated positive findings in BD, including decreased cortical thickness and glial density in subgenual anterior cingulate cortex, reduced neuronal density in some amygdalar nuclei, and decreased calbindin-positive neuron density in prefrontal cortex. Many other positive findings have also been reported, but with limited or contradictory evidence. As an important negative result, it can be concluded that gliosis is not a feature of BD; neither is there neuropathological evidence for an inflammatory process.

## Introduction

Like other ‘functional’ psychiatric disorders, bipolar disorder (BD) lacks any diagnostic neuropathology of the kind which characterises and defines the dementias, but this does not mean that BD has no morphological correlates. Magnetic resonance imaging (MRI) studies show small but robust differences in the volumes of some brain structures, notably decreases in hippocampus, amygdala and thalamus, and reduced cortical thickness [1, 2]. There is also increasing evidence for white matter decrements [3, 4] with anatomical and functional dysconnectivity of specific pathways and circuits [5, 6]. Presumably these neuroimaging findings are reflected in alterations at the histological and cellular level. However, a review covering the period up to 1999 noted the remarkable lack of data [7]. By the definition of neuropathology adopted here (see Methods and Materials), the literature at that time comprised only nine publications. Vawter and colleagues [7] drew attention to some preliminary findings, notably a report of decreased glial density in the subgenual anterior cingulate cortex (sgACC) in BD and major depressive disorder [8], and a pilot study describing decreased interneuron density in the hippocampal CA2 subfield [9].

Öngür et al. (1998) [8] remains by some distance the most cited paper on the neuropathology of BD (Supplementary Table 1), and it was soon followed by a series of other morphometric studies. Many took advantage of the brain series collected by the Stanley Foundation (subsequently the Stanley Medical Research Institute), called the Stanley Neuropathology Consortium [10]. For the first time, this provided brain tissue specifically designed to allow comparison of BD, schizophrenia and major depressive disorder with healthy comparison subjects (*n* = 15 in each group) [10]. Moreover, tissue was provided blind to diagnosis such that researchers had to include BD, even if their primary interest lay in one of the other diagnoses.

An updated narrative review of the neuropathology of mood disorder was reported by Harrison (2002) [11], by which time the BD literature extended to 27 papers. More recently, Price and Drevets (2010) [12] reviewed mood disorder neuropathology in the context of neuroimaging findings and normative brain connectivity, and Savitz et al. (2014) [13] focused on the prefrontal cortex. To our knowledge, there has been no substantive review covering BD neuropathology since then, and there has never been a systematic review. Here, we report the latter, accompanied by meta-analyses where possible. The review was registered on the PROSPERO international prospective register of systematic reviews (CRD42018089740).

## Methods and Materials

### Scope of systematic review

We adopted a pragmatic definition of neuropathology to comprise studies which measured ‘visible’ parameters such the density, number, size or shape of cells (neurons, glia or subpopulations thereof), cellular constituents (e.g. synapses, dendrites and mitochondria), or cytopathological elements (e.g. neurofibrillary tangles, amyloid plaques), in patients with BD compared to control subjects. We included studies which identified neuronal and glial populations using antigens generally recognised for this purpose (e.g. parvalbumin [PV], glial fibrillary acidic protein [GFAP], ionised calcium-binding adaptor molecule 1 [Iba-1]). We also included studies which measured the size of a brain structure (e.g. cortical thickness, hippocampal volume). We excluded studies which used messenger RNAs to delineate cell populations or which used proteins as proxy markers of sub-cellular compartments (e.g. synaptophysin as a marker of presynaptic terminals, or spinophilin for dendritic spines). We also excluded studies using brain homogenates.

### Literature search and data extraction

We identified publications by searching the Web of Science Core Collection (1945 to 8th June 2018) and MEDLINE (1950 to 8^th^ June 2018) using the following search terms: (‘bipolar disorder’ or ‘bipolar affective’ or ‘bipolar illness’ or ‘bipolar disease’ or ‘manic-depressi*’ or ‘manic depressi*’) and (‘neuropatholog*’ or ‘morphometr*’ or ‘neuron*’ or ‘glia*’ or ‘pyramidal’ or ‘oligodendro*’ or ‘astrocyt*’ or ‘astrogli*’ or ‘microgli*’ or ‘*gliosis’). We also searched papers’ reference lists and PJH’s reprint collection to identify additional studies meeting our criteria. We did not consider papers including less than three BD cases, conference abstracts, non-peer reviewed publications (e.g. book chapters), nor data papers not published in English. Two authors (PJH, with either LC or CHH) independently conducted the searches and the data extraction, and all authors met to resolve any divergent results. Graphical data were extracted using Webplotter (https://apps.automeris.io/wpd/).

We decided a priori to meta-analyse studies where at least two datasets were available, and which had measured the same parameter in the same brain region. In practice, this required judgement about what constituted meta-analysable data. Only data presented in the form of group means together with a measure of variance were considered for meta-analysis; this excluded several studies (e.g. which used medians and interquartile ranges). Meta-analyses were conducted with a fixed effects model, using Review Manager (RevMan) 5.3. Standardised mean differences (SMD) were used except where stated. Where necessary, standard errors were converted to standard deviations. If subgroups needed to be combined for meta-analysis (e.g. if a paper analysed males and females separately, or presented data for both right and left hemisphere), we used the formulae for weighted means and standard deviations in the Cochrane Handbook [14]. For studies reporting data for individual cortical layers or hippocampal subfields, a summary statistic for an overall diagnostic effect across all layers/subfields was also computed by RevMan; this statistic uses the number of observations not the number of subjects to calculate significance, and so should be interpreted with caution.

## Results

The literature search found 5388 publications meeting our criteria, and an additional 15 papers were found from other sources. Of the 5403 papers, 103 were selected for detailed inspection based on the abstract. 81 proved to have eligible original data [8, 9, 15–93], and form the focus of the systematic review. The 22 excluded papers are listed in Supplementary Table 2 together with the reason for their omission. The PRISMA diagram is shown as Supplementary Figure 1.

### Characteristics of included studies

The basic demographics of the 81 studies are shown in Table 1.

**Table 1:** Demographic characteristics of the 81 studies included in the systematic review

	Mean	SD	Range
**BD subjects per study** (*n*)^a^	11.5	4.3	3–21
Females	5.4	2.5	1–12
Males	6.4	2.7	1–10
**Controls per study** (*n*)^a,b^	14.8	7.0	3–55
Females	6.3	4.0	1–33
Males	8.7	3.6	2–24
**Ages (years)**			
BD	50.6	9.7	27–74
Controls	54.0	7.8	42–75

^a^Sex-specific information known for *n* = 78 studies.

^b^Excluding Shioya et al. [89]; see Table 6.

Most studies utilised tissue from recognised brain collections, notably the Stanley Neuropathology Consortium (*n* = 35 studies), Harvard/McLean brain bank (*n* = 17), and Magdeburg brain collection (*n* = 11). The remainder came from a range of other sources; some studies used more than one series. Most studies used diagnostic criteria for BD: DSM-IV (*n* = 59), DSM-III-R (*n* = 13), and ICD-10 (*n* = 4); the remaining studies used other criteria or did not specify. Reflecting the fact that the brain collections mentioned above also include schizophrenia and/or major depressive disorder, many studies also included one or both diagnostic groups (schizophrenia [*n* = 58], major depressive disorder [*n* = 57]). In terms of anatomical focus, a range of brain regions have been studied: prefrontal cortex (PFC; *n* = 19), especially dorsolateral (DLPFC; *n* = 15); ACC, including sgACC (*n* = 17); other neocortical regions (*n* = 10); hippocampus (*n* = 13); amygdala (*n* = 8); entorhinal cortex (*n* = 7); white matter (*n* = 10); thalamus (*n* = 6); other (*n* = 18). Fourteen studies included more than one region. We rated 28 studies as adhering broadly to stereological principles (e.g. random sampling, 3D counting), 24 did so partially, and 29 either did not or could not be rated. Sixty five studies were carried out blind to diagnosis; no information about blinding was given for 16 studies.

**Fig. 1:**
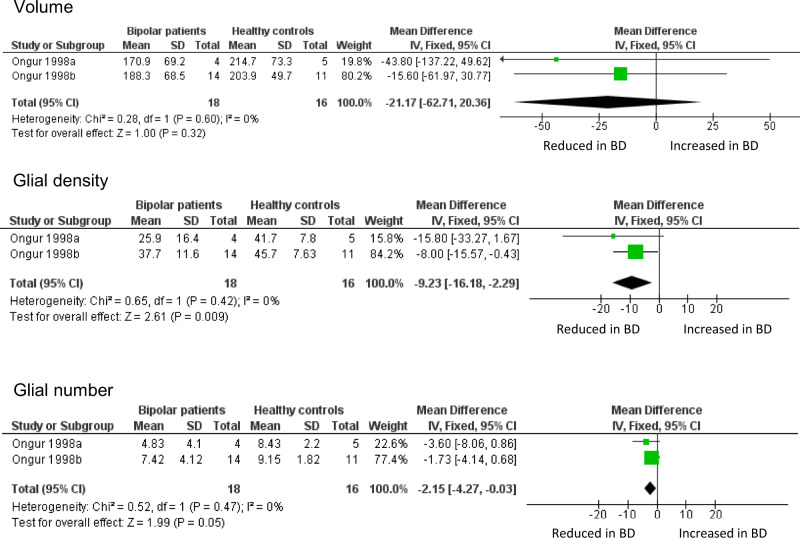
Meta-analysis of volume, glial density, and glial number in subgenual ACC. Data are taken from the two cohorts included in Öngür et al. [8]. ‘Ongur 1998a’ refers to their pilot study; ‘Ongur 1998b’ refers to the main study, which used brains from the Stanley Foundation. Results are presented as mean differences. Glial density (cells/mm^3^ x 10^3^) is reduced, with a borderline reduction in glial number (x10^6^), but no difference in sgACC volume (mm^3^)

**Fig. 2:**
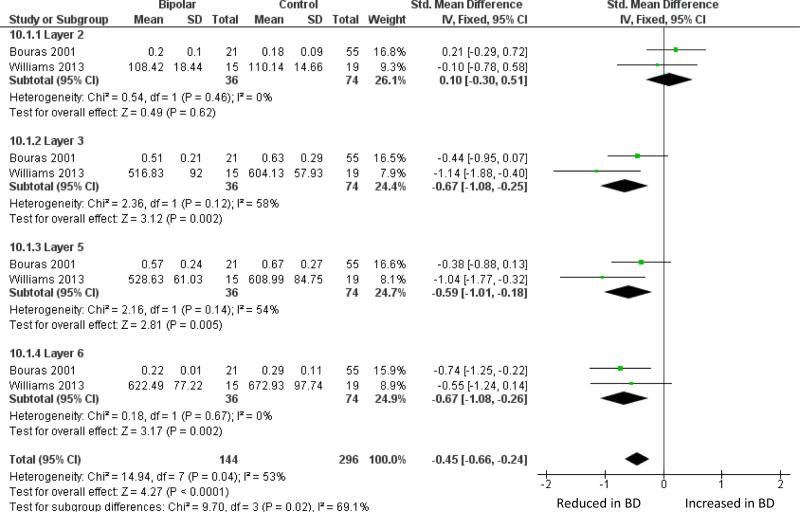
Meta-analysis of layer thickness in subgenual ACC. Meta-analysis of Bouras et al. [26] and Williams et al. [82] reveals decreased thickness in BD of sgACC layer III (SMD −0.67, *p* = 0.002), layer 5 (SMD −0.59, *p* = 0.005) and layer VI (SMD −0.61, *p* = 0.004). Thickness is also decreased across all four layers considered together (SMD −0.45, *p* < 0.0001). The data in ref. 82 were originally analysed separately for men and women; they are combined here as described in the text

**Fig. 3:**
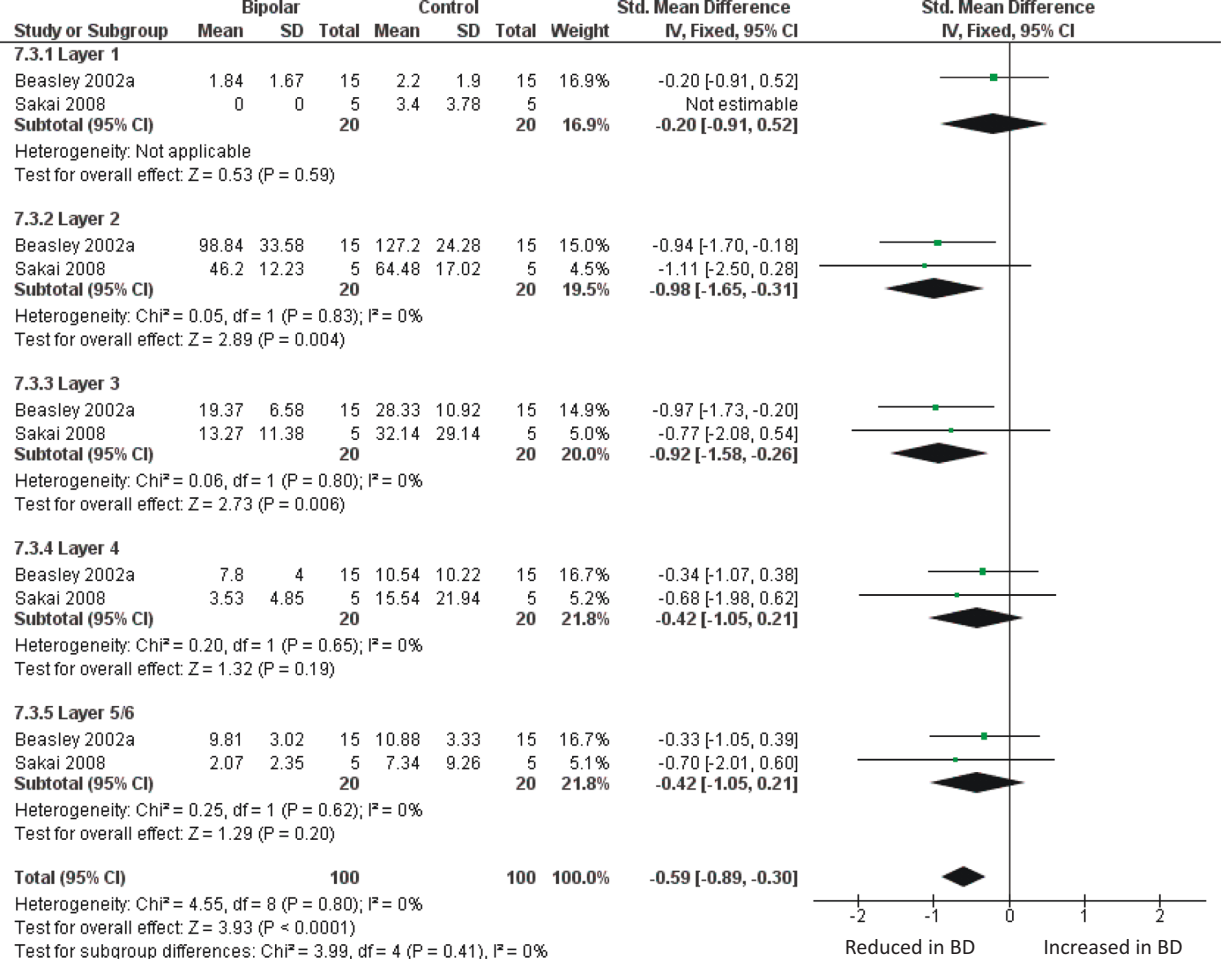
Meta-analysis of calbindin-immunoreactive neuron density in DLPFC. Meta-analysis of Beasley et al. [32] and Sakai et al. [62]. We used the data for the ‘medium’ size class of neuron reported in ref. 62. Density of CB-positive neurons is reduced in BD in layer II (SMD −0.98, *p* = 0.004) and layer III (SMD −0.92, *p* = 0.006), and with an overall significant reduction if all 5 layers are considered together (SMD −0.59 *p* < 0.0001)

**Fig. 4:**
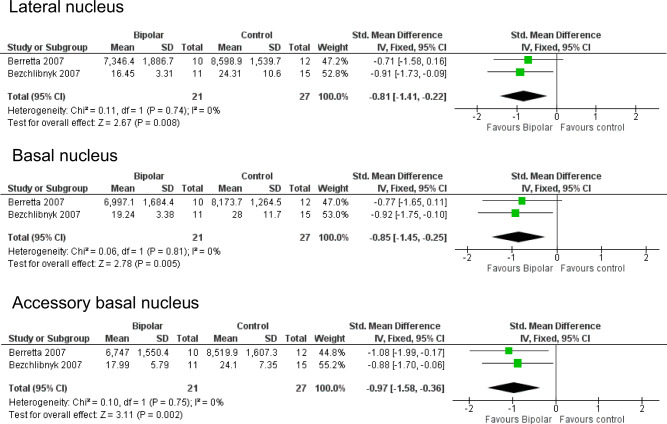
Meta-analysis of neuronal density in lateral, basal and accessory basal nuclei of the amygdala. Meta-analysis of Berretta et al. [53] and Bezchlibnyk et al. [54]. For the accessory basal nucleus, we combined data from parvocellular and magnocellular subnuclei reported in [54]. Neuronal density is reduced in BD in the lateral nucleus (SMD −0.81, *p* = 0.008), basal nucleus (SMD −0.85, *p* = 0.005) and accessory basal nucleus (SMD −0.97, *p* = 0.002). SMDs should be interpreted taking into account the fact that ref. 53 used 3D counting (neurons per unit volume) whereas ref. 54 used 2D counting (neurons per unit area)

Results are discussed region by region, and summarised in Tables 2–6 and Supplementary Tables 3 and 4. The Tables highlight, for each study, the main results which the authors reported as being statistically significant, as well as important negative findings. Studies which we meta-analysed and their findings are described in the text, summarised in Supplementary Table 5, and illustrated in Figs. 1–4 and Supplementary Figures 2–7. In the event, only two datasets for any given parameter in any one brain region were amenable to meta-analysis.

**Table 2:** Studies of anterior cingulate cortex

Study	Sample (Con/BD)	Sub-regions investigated	Stain	Parameters measured	Key significant findings in BD
Öngür et al. (1998) [8]	A:5/4 B:11/14	Subgenual	Nissl	Volume. Neuronal and glial number, density and size.	A: Decreased glial density. B: Decreased glial density^a^. No differences in volumes or neuronal parameters.
Benes et al. (2001) [25]	12/10	Pregenual, BA24, layers I-VI	Nissl	Layer thickness. Density and size of pyramidal and non-pyramidal neurons. Density of glia.	Layer I larger (+21%), layer III smaller (−12%). Non-pyramidal neurons: density decreased in layer II (−28%), size increased in layer II (+21%) and III (+23%). Pyramidal neurons larger in layer II (+24%). No differences in glial density or pyramidal neuron density.
Bouras et al. (2001) [26]	55/21	Subgenual and supragenual (BA24a/b), layers II, III, V, VI	Nissl	Cortical and layer thickness. Neuronal density and size.	Subgenual: reduced cortical thickness (−20%), significant in layers III, V, and VI (all −15–21%). Decreased neuronal density in layer III (−31%), V (−25%) and VI (−23%). No difference in neuronal size. Supragenual: no differences.
Cotter et al. (2001) [27]	15/15	Supragenual, BA24b, layers I, II, III, Va, Vb, VI	Nissl	Neuronal and glial density. Neuronal size.	No differences.
Cotter et al. (2002b) [36]	15/15	Supragenual, BA24	CB, CR, PV	Neuronal density. Neuronal size. Clustering of neurons.	Reduced density of CB-positive neurons in layer II (−33%).^b^ Increased clustering of PV-positive neurons. No differences in CR-positive neurons.
Chana et al. (2003) [40]	15/15	Supragenual, BA24c	Nissl	Layer thickness. Neuronal and glial density. Neuronal and glial size. Clustering of neurons and glia.	No difference in cortical layer thickness. Neuronal density increased in layer VI (+56%). Neuronal size reduced in layer III^b^ and V. Reduced neuronal clustering. No glial differences.
Todtenkopf et al. (2005)^c^ [50]	6/6	Pregenual, BA24, layers I-VI	Nissl	Density of pyramidal and non-pyramidal neurons, and glial density.	Reduced pyramidal neuron density in layer V (−12%). Reduced non-pyramidal neuron density in layers V (−12%) and VI (−21%). Reduced glial density in layers III (−35%), V (−37%) and VI (−31%).
Bielau et al. (2007) [55]	19/11	Pregenual	GAD	Neuronal density	No differences
Connor et al. (2009) [64]	45^d^/15	Supragenual, caudal BA24 and underlying white matter, compartments I–V	NeuN, Iba-1	Neuronal density. Neuronal size. Iba-1-positive microglial density in white matter.	Neuronal density increased (+79%) in white matter, significant in compartments II–V. Neuronal density unchanged in grey matter (*n* = 5 cases). No differences in neuronal size. No differences in Iba-1 immunoreactivity.
Brüne et al. (2010) [66]	22/20	Supragenual, BA24, layer Vb	Nissl	Cortical and layer thickness. Density of VENs.	No differences
Hercher et al. (2010) [68]	7/5	Supragenual, BA24, layer VI	Golgi	Dendritic branching	Fewer third-order branches
Sinka et al. (2012) [78]	7/10	Subgenual and dorsal BA24, layers III and V	Nissl, Gallyas	Cortical and layer thickness. Neuronal density. Diameter of capillaries.	No difference in cortical or layer thickness. Neuronal density increased in dorsal BA24 layers III (+29%) and V (+32.3%). Reduced capillary diameter in both layers in dorsal and subgenual ACC (overall −23%).
Mosebach et al. (2013) [81]	16/8	Pregenual, BA32, and underlying white matter	Nissl, olig1	Oligodendrocyte density	No differences
Williams et al. (2013a) [82]	19/15	Subgenual	Nissl	Cortical and layer thickness	Cortex thinner at crown (−10%). No changes in layer thickness^e^
Williams et al. (2013b) [83]	19/15	Subgenual	Nissl, GFAP	Density of GFAP-positive cells. Density of oligodendrocytes	No differences
Williams et al. (2014) [86]	19/15	Subgenual	GFAP	Density of fibrillary and gemistocytic astrocytes	No differences
Krause et al. (2017) [91]	3/3	Supragenual, BA24b, layer Vb	EM	Lysosomal aggregations in VENs and pyramidal neurons	No differences

^a^One-tailed test used. Other analyses significant when familial BD (*n* = 5) considered separately (see text).

^b^After Abercrombie correction; stated as non-significant when corrected for multiple testing for 6 layers.

^c^Only data from ‘Study D’ included.

^d^*n* = 21 for Iba-1.

^e^In sgACC, layer V was thinner in BD in the right hemisphere, and there was a layer thickness x sex interaction with a trend (*p* = 0.086) reduction in layer III in male BD cases.

*BA*: Brodmann area. *CB*: calbindin. *CR*: calretinin. *EM*: electron microscopy. *GAD*: glutamic acid decarboxylase (65 and 67 kDa forms). *Iba-1*: ionized calcium-binding adaptor molecule 1. *NeuN*: neuron-specific nuclear antigen. *PV*: parvalbumin. *VENs:* von Economo neurons.

**Table 3:** Studies of prefrontal cortex

Study	Sample (Con/BD)	Sub-regions investigated	Stain	Parameters measured	Key significant findings in BD
Guidotti et al. (2000) [23]	15/15	DLPFC (BA9), layer I	Nissl, reelin	Density of neurons	Density of reelin-positive neurons decreased (−28%). Overall neuronal density unchanged.
Rajkowska et al. (2001) [24]	11/10	DLPFC (BA9), layers I, II, IIIa-c, IV, Va,b, VI	Nissl	Cortical and layer thickness. Density, size, and shape, of neurons and glia.	Sublayer IIIc thicker (16%). Decreased neuronal density in layer IIIa-c (−12–23%). Decreased density of pyramidal neurons in layers IIIa-c (20–32%) and Va (−27%). Decreased density of ‘medium-sized neurons’ (−35%). Decreased glial density in layer IIIc (−19%) and Vb (−12%). Glia enlarged in layer I ( + 9%) and IIIc ( + 7%).
Uranova et al. (2001) [29]	16/6	Frontal pole (BA10), layer VI	EM	Oligodendroglial ultrastructure	Apoptosis and necrosis noted. No quantitative data presented.
Webster et al. (2001) [30]	15/15	Middle frontal gyrus	pGFAP	Frequency and distribution of pGFAP-positive cells	Semi-quantitative study. No differences.
Beasley et al. (2002a) [32]	15/15	DLPFC (BA9), layers I, II, III, IV, V/VI.	CB, CR, PV	Cortical thickness. Density of neurons.	Density of CB-neurons decreased in layers II (−22%) and III (−32%).^a^ No other significant differences.
Beasley et al. (2002b) [33]	15/15	DLPFC (BA9/46), white matter compartments I-V	MAP-2	Density, size, and clustering of MAP-2-positive neurons.	No differences.
Cotter et al. (2002b) [36]	15/15	DLPFC (BA9), layers I-VI	Nissl	Density, size, and clustering of neurons and glia.	Glial density reduced in layer VI (−7%).^a,b^ Neuronal size decreased in layer III (−10%)^a,b^, V (−14%)^b^and VI (−25%)^b^. No differences in cell density or clustering.
Law and Harrison (2003) [41]	15/15	DLPFC (BA9), layers III and V	SMI32	Density, size and shape of SMI32-positive neurons	No differences.
Uranova et al. (2004) [44]	15/15	DLPFC (BA9), layer VI and white matter	Nissl	Cortical layer thickness. Density of oligodendroglia.	Decreased oligodendroglial density in layer VI (−29%). No difference in white matter.
Cotter et al. (2005) [48]	15/14	Caudal OFC, layers I-VI	Nissl	Cortical layer thickness. Density and size of neurons and glia.	Decreased neuronal size in layer I (−21%)^b^ and V (−20%)^a,b^.
Toro et al. (2006) [52]	15/15	DLPFC (BA9) and OFC (BA11/47)	GFAP, GS	Immunoautoradiography.	Reduced GFAP signal in orbitofrontal cortex (−13%). No change in GS.
Bielau et al. (2007) [55]	19/11	DLPFC and OFC	GAD	Density of GAD-positive neurons.	Increased density in OFC ( + 133%).
Vostrikov et al. (2007) [58]	15/15	DLPFC (BA9), layer IIIa,b,c	Nissl	Size of pyramidal neurons. Density of perineuronal oligodendroglia.	Decreased pyramidal neuron size in layer IIIc (−4%). Fewer perineuronal oligodendroglia in layers IIIa (−29%), IIIb (−40%) and IIIc (−49%).
Sakai et al. (2008) [62]	5/5	DLPFC (BA9), layers I, II, III, IV, V/VI	Nissl, CB, CR, PV	Density and size of neurons and glia.	Decreased neuronal density overall and in layer IV (−28%).^c^ Increased density of ‘large’ CR-positive neurons in layer II ( + 209%).
Cataldo et al. (2010) [67]	10/10	Prefrontal cortex	EM	Size of neuronal mitochondria.	Mitochondria smaller.
Mosebach et al. (2013) [81]	16/8	DLPFC (BA9), grey and white matter	Nissl, olig1	Density of oligodendroglia.	No differences.
Hercher et al. (2014) [84]	20/20	DLPFC (BA9), white matter	Nissl, GFAP, Iba-1	Density of cells. Size of nuclei. Clustering of cells. Area fraction occupied by GFAP- or Iba-1-positive cells.	Increased density of oligodendroglia (+17%). Decreased GFAP area fraction (−17%). Increased clustering of GFAP-positive astrocytes. No change in Iba-1.
Konopaske et al. (2014) [85]	19/9	DLPFC (BA46), deep layer III pyramidal neurons	Golgi	Dendritic spine density. Spines per dendrite. Dendritic length.	Decreased spine density (−10%). Fewer spines per dendrite (−26%). Decreased dendritic length (−19%).
Bernstein et al. (2015) [87]	16/15	DLPFC (BA9)	GS	Density of GS-positive astrocytes and GS-positive oligodendrocytes	No differences

*EM*: electron microscopy. *GS*: glutamine synthetase. *MAP-2*: microtubule-associated protein 2. *OFC*: orbitofrontal cortex. *pGFAP*: phosphorylated glial fibrillary acidic protein. *SMI32*: antibody against neurofilament proteins, labelling corticocortical projection neurons.

For other abbreviations see earlier Tables, and text.

^a^Noted as being non-significant when Bonferroni-corrected.

^b^Percent differences in medians.

^c^Noted as being non-significant after Abercrombie correction.

**Table 4:** Studies of amygdala

Study	Sample (Con/BD)	Amygdalar nuclei investigated	Stain	Parameters measured	Key significant findings in BD
Bowley et al. (2002) [34]	10/12	Not differentiated	Nissl	Neuronal and glial density	No differences
Hamidi et al. (2004) [43]	10/9	Not differentiated	Nissl, S-100β, HLA	Glial density	No differences
Bielau et al. (2005) [47]	22/11	Not differentiated	Nissl, myelin	Volume	No differences
Berretta et al. (2007) [53]	12/10	Lateral, basal, accessory basal and cortical	Nissl	Volumes. Neuronal number, density and size.	Lateral nucleus: reduced volume (−29%), neuron number (−41%) and density (−14.5%). Accessory basal nucleus: reduced neuronal density (−21%). No other differences.
Bezchlibnyk et al. (2007) [54]	15/11	Lateral, basal, and accessory basal (parvocellular and magnocellular)	Nissl	Neuronal and glial density. Neuronal and glial size.	Reduced neuronal size in lateral (− 29.7 %) and accessory basal parvocellular (− 28.3 %) nuclei. No differences in cell densities.
Altshuler et al. (2010) [65]	14/10	Basolateral	Nissl, GFAP	Density of neurons and GFAP-positive astrocytes.	No differences
Pantazopoulos et al. (2010) [70]	15/11	Lateral, basal, accessory basal and cortical	GFAP, PV	Number and density of PV-positive neurons. Number and density of GFAP-positive astrocytes.	No differences
Pantazopoulos et al. (2017) [92]	15/15	Lateral, basal, accessory basal and cortical	NPY, SS	Volumes. Number and density of NPY- and SS-positive neurons.	Decreased volume of lateral (− 22%) and cortical (− 31%) nuclei. SS-positive neurons: decreased number (− 26%) and density in lateral nucleus. NPY-positive neurons: decreased number in cortical nucleus (− 29%). No other differences.

*NPY*: neuropeptide Y. *SS*: somatostatin.

**Table 5:** Studies of hippocampus

Study	Sample size (Con/BD)	Regions of hippocampus	Stain	Parameters measured	Key significant findings in BD
Benes et al. (1998) [9]	11/4	Middle; CA1,2,3,4	Nissl	Neuronal density, neuronal size, area of subfields	Lower density (−56%) and number (−60%) of non-pyramidal neurons in CA2; size of these neurons reduced (−12%). Pyramidal neurons unaffected. Subfield areas unchanged.
Fatemi et al. (2000) [22]	15/15	Posterior; various strata and subfields	Nissl, Reelin	Reelin + cell density, area of subfields	Reduced density in DG (−32%) and CA4 (−36%).
Webster et al. (2001) [30]	15/15	Hilus, pial surface	pGFAP	Frequency of pGFAP + cells	No differences
Damazdic et al. (2002) [37]	11/8	Anterior	Silver	Neurofibrillary tangles, senile plaques	No differences
Gilmore et al. (2002) [38]	15/15	Anterior; ependyma	H&E	Discontinuties; nodular gliosis; subventricular rosettes	No differences
Zhang and Reynolds (2002) [39]	15/14	Middle; DG, CA1,2,3,4	PV,CR	Neuronal density, neuronal size, area of subfields	Reduced density of PV + neurons in all subfields (−35%). Reduced size of PV + neurons. No differences in CR + neurons, or subfield areas (data not shown).
Bielau et al. (2005) [47]	22/11		Nissl	Hippocampal volume	Decreased in BD (−9.5%; effect size 0.36).
Bielau et al. (2007) [55]	19/11		GAD	Neuronal density	No differences.
Liu et al. (2007) [56]	14/14	Anterior; CA1	Nissl	Neuronal size	Reduced (−12%).
Konradi et al. (2011) [73]	18/14	Whole; CA1, 2/3,4	Nissl, PV, SS	Volume of subfields (pyramidal and non-pyramidal strata). Neuronal number and size.	Reduced volume of non-pyramidal strata (−14%). Reduced soma volume (−18%) in CA2/3. Fewer PV + neurons overall, significant in CA1 (−31%) and CA4 (−46%). Fewer SS + neurons overall, significant in CA1 (−43%).
Wang et al. (2011) [74]	16–17/9–13	Pre- and para-subiculum, subiculum	PV,SS,CB	Neuronal density	Reduced density of SS+ and PV+ neurons in parasubiculum. No change in CB+ neurons.
Gos et al. (2013) [80]	12/6	Posterior	GFAP	Cell density (stratum pyramidale and alveus)	No differences.
Malchow et al. (2015) [88]	10/8	Posterior; CA1,2/3,4, subiculum	Nissl	Subfield volumes. Number and density of neurons, oligodendrocytes and astrocytes	No differences in volumes. Neuronal number increased in CA1 (+34%) and subiculum (+36%). Neuronal density increased in CA4 (+19%) and subiculum (+33%). Oligodendrocyte number increased in CA1 (+27%). Astrocytes unaffected.

**Table 6:** Studies of subcortical regions

Study	Sample size (Con/BD)	Region(s) investigated	Stain	Parameters measured	Key significant findings in BD
Nasrallah et al. (1983) [16]	11/7	Corpus callosum	Nissl, silver	Glial density	No differences.
Purba et al. (1996) [18]	8/3	Hypothalamus: PVN	AVP, OXT	AVP- and OXT-positive neuron number.	AVP neuron number increased (+64%).^a^
Baumann et al. (1999a) [19]	12/6	Locus ceruleus	Nissl	Size; number and density of melanin-positive neurons	Neuron number increased (+10%), significant in rostral (+39%) and medial (+14%) regions. Increased number of large neurons.
Baumann et al. (1999b) [20]	12/6	Locus ceruleus	TH	TH-positive neuron density	No differences
Helmkamp et al. (1999) [21]	15/12	Cerebellum: vermis	Nissl	Purkinje cells: density, proportion of displaced cells	No differences
Uranova et al. (2001) [29]	16/6	Caudate nucleus	EM	Oligodendroglial ultrastructure	‘Signs of apoptosis and necrosis’. No quantitative data presented.
Baumann et al. (2002) [31]	12/6	Dorsal raphe nucleus	Nissl	Number of neurons. Volume of subnuclei.	Fewer neurons in ventral subnuclei of rostral dorsal raphe.
Young et al. (2004) [45]	11/13	Thalamus: mediodorsal and AVM nuclei	Nissl	Volume of nuclei. Number and density of neurons.	No differences.
Bielau et al. (2005) [47]	22/11	Basal ganglia, thalamus, hypothalamus, diencephalon, subcortical grey matter	Nissl, myelin	Volume	Decreased volume of: basal ganglia (−9.8%), putamen (−10.1%), pallidum externum (−8.0%) and internum (−9.3%); thalamus (−10.9%); hypothalamus (−15.5%); diencephalon (−11.3%); subcortical grey (−9.9%).
Manaye et al. (2005) [49]	8/7	Hypothalamus: PVN and SON	Nissl	Volume. Neuron number.	Number of PVN neurons reduced (~ −50%). No difference in SON. No difference in volumes of PVN or SON.
Bielau et al. (2007) [55]	19/11	Thalamus: mediodorsal nucleus	GAD	GAD-positive neuron density	No differences
Young et al. (2007) [59]	15/11	Thalamus: pulvinar nucleus	Nissl	Volume. Neuron number.	Pulvinar volume increased ( + 12%), though data not presented. No difference in neuron number.
Byne et al. (2008) [60]	14/15	Thalamus: anterior and centromedian nuclei	Nissl	Volume. Number of neurons and oligodendrocytes.	No differences. Post hoc, reduced oligodendrocyte number in both nuclei (~ 45%) in BD cases with psychosis.
Maloku et al. (2010) [69]	24/17	Cerebellum: cortex	Nissl	Linear density of Purkinje cells. Density of granule cells.	Reduced density of Purkinje cells (−20%). No differences in granule cell density.
Ranft et al. (2010) [71]	13/8	Habenula: medial nucleus	Nissl	Neuronal density	No differences (data not shown)
Brisch et al. (2011) [72]	14/8	Septal nuclei	Nissl, myelin	Area of nuclei. Density and size of neurons.	Reduced neuron density in lateral septal nucleus. No other differences.
Bernstein et al. (2012) [75]	20/10	Mammillary bodies. Fornix.	Nissl, myelin	Volumes. Density and number of neurons.	Mammillary body volume reduced (−24%). No other differences.
Comte et al. (2012) [76]	15/15	Lateral ventricle: SVZ and ependymal	Nissl, MHCII, AcTub	Density of neurons in hypocellular gap. Density of ependymal cells. Width of hypocellular gap.	No differences.
Matthews and Harrison (2012) [77]	13/13	Dorsal raphe nucleus	NeuN, PH8	Area. Density and size of neurons.	Reduced size of PH8-positive neurons in rostral dorsal raphe (−24%). No other differences.
Gao et al. (2013) [79]	12/5	Hypothalamus: PVN	CRH	Number of neurons	Increased 64%.
Williams et al. (2013b) [83]	19/15	Corpus callosum, genu	Nissl, GFAP	Density of oligodendrocytes and astrocytes	No differences
Bernstein et al. (2015) [87]	16/15	Nucleus accumbens	GS	Density of astrocytes and oligodendrocytes	No differences
Shioya et al. (2015) [89]	1240/11^b^	Multiple	Various	Semi-quantitative ratings of neurodegenerative features	8 BD cases met criteria for a neurodegenerative disorder. Younger cases had more argyrophilic grains than expected.
Brisch et al. (2017) [90]	22/5	Dorsal raphe nucleus	HLA-DR	Density of activated microglia	No differences
Steullet et al. (2018) [93]	16/15	Thalamus: reticular nucleus	Nissl, PV	Volume. Number and density of PV-positive neurons.	Decreased volume of reticular nucleus (−22%). Decreased number (−10%) and density (−15%) of PV-positive neurons.

*AcTub*: acetylated tubulin. *AVM*: anteroventral/anteromedial. *AVP*: arginine vasopressin. *CRH*: corticotropin-releasing hormone. *GS*: glutamine synthetase. *OXT*: oxytocin. *PH8*: phenylalanine hydroxylase (marker of 5-HT neurons). *PVN*: paraventricular nucleus. *SON*: supraoptic nucleus. *SVZ*: subventricular zone. *TH*: tyrosine hydroxylase.

For other abbreviations see preceding Tables, and text.

^a^BD and MDD data analysed together in the paper. Comparison restricted to BD (*n* = 3) vs controls shows increased AVP neuron number (*p* = 0.012) but no difference in OXT neuron number (*p* = 0.19; Mann–Whitney test).

^b^11 consecutive BD cases compared with up to 1240 other subjects. No formal case–control comparisons made.

### Anterior cingulate cortex (ACC)

The ACC is an anatomically and functionally heterogeneous structure at the interface of cognition, emotion and behaviour [94–96]. It became of great interest in mood disorders after a study showing a focal decreased volume, and reduced metabolism, in the sgACC [97]. The same group then sought an anatomical correlate of these findings. They reported (in a pilot study and in a second, larger cohort) a reduction of glial density, using unbiased stereological methods on Nissl-stained sections, present in BD and in subjects with major depressive disorder [8]. Neuronal density and number were unchanged, and sgACC volume non-significantly reduced. Glial density was unaltered in the parietal cortex, suggesting a degree of anatomical localisation, and glial density was unchanged in the sgACC in schizophrenia, indicating a degree of diagnostic specificity.

Öngür et al. [8] was arguably the first significant neuropathological study of BD, and the first to use contemporary methods. It has been followed by 16 further studies of ACC neuropathology in BD (Table 2); 4 include sgACC, 13 examined other parts of the ACC. However, there have been no direct replications of the design or methods used by Öngür et al., precluding any meta-analysis of their data beyond simply combining the two datasets in their original paper. This confirms the reduction in glial density in BD in sgACC, and also shows a borderline significant decrease in glial number, but no difference in sgACC volume (Fig. 1). The findings strengthen the conclusions drawn by Öngür and colleagues, not least since their paper used one-tailed tests for some analyses.

The largest BD ACC study is by Bouras et al. [26], who reported reduced neuronal density in layers III, V, and VI of sgACC. These laminae were also thinner than in controls, as was the grey matter as a whole. The changes were similar but less pronounced in schizophrenia, and not seen in major depression. They state that no differences between BD and controls were seen in occipital cortex, but do not present the data. Glia were not measured. In a much smaller study of sgACC, Sinka et al. [78], who only measured layers III and V, found a non-significant trend towards decreased thickness of layer III, but no difference in neuronal density in BD in either lamina. Again, glia were not measured. Williams et al. [82] found a thinner grey matter in the sgACC crown in BD, with a reduced layer V thickness in the right hemisphere. Meta-analysis of sgACC layer thickness using data from refs. 26 and 82 shows a decrease in layers III, V, and VI in BD (Fig. 2). In a companion paper, Williams et al. (2013b) [83] counted sgACC oligodendrocytes using a Nissl stain, and astrocytes using GFAP immunohistochemistry, and found no differences in BD.

Studies of the non-subgenual parts of ACC provide a mixed picture (Table 2). Bouras et al. [26] measured the same parameters as noted above in sgACC, and found no differences between BD and controls in the dorsal ACC. However, when their layer thickness data were meta-analysed together with Benes et al. [25], the trend reduction seen in both studies for a thinner layer V became significant (Supplementary Figure 2).

The main finding of Benes et al. [25], who studied the rostral ACC, was a marked reduction in the density of non-pyramidal neurons in layer II, which remained significant after Abercrombie correction for cell size. The latter point is relevant since the neurons were larger in BD than in controls. The authors found no differences in pyramidal neuron density, or in glial density, in BD. The same group later used a different (three-dimensional, stereological) counting method in a subset of the same brains, and found modest but significant reductions in the density of non-pyramidal and pyramidal neurons, and glia, in BD, mostly in layer V [50]. In contrast, Cotter et al. [27] found no differences in glial density, neuronal density, or neuronal size in BD in the supra-callosal ACC (Brodmann area [BA] 24b) using a stereological approach. This group later used an adjacent part of the supragenual ACC (BA24c) of the same subjects for a two-dimensional study of density, size, and clustering of neurons and glia; in BD, neuronal size was decreased in layer V, and neuronal density increased in layer VI [40]. Neither study could be included in a meta-analysis because data were presented as medians [27] or as means but without any measure of variance [40].

Connor et al. [64] studied the white matter below the caudal ACC to study the density of white matter neurons (stained by NeuN) since altered distribution of these neurons in schizophrenia had been reported, and viewed as indicative of disordered neurodevelopment. In this relatively large study, they reported an increased density of ACC white matter neurons in BD, with a similar finding in PFC white matter.

In summary, there have been intriguing findings in the sgACC in BD, notably the glial deficits identified by Öngür et al. [8] and a thinning of the cortex found in two independent studies [26, 82]. In other regions of the ACC, the findings are less prominent and not well replicated for either glial or neuronal alterations, though there is moderate evidence for a thinner layer V.

### Prefrontal cortex (PFC)

The PFC has been the most studied brain region in BD. Virtually all studies have been carried out in the DLPFC (BA9 and 46). This neuropathological focus can be traced in part to the prominence of this region in studies of schizophrenia at the time when the Stanley Neuropathology Consortium tissue was being made available (see Introduction) [98, 99]. There had also been emerging interest in cognitive aspects of BD which suggested potential involvement of the PFC [100, 101].

Neuropathological studies of PFC in BD are summarised in Table 3. The first report was by Guidotti et al. (2000) [23]. Amongst other parameters, they reported a marked decrease in the density of reelin-positive neurons in layer I (wherein most such cells are located) of BA9, with no change in overall neuronal density. The first dedicated, three-dimensional counting study of PFC was by Rajkowska et al. [24], again in BA9. Neuronal density was reduced in layer III, and pyramidal neuron density reduced in layers III and V. Glial density was decreased in layer III, with glial size increases. These authors noted the similarity of the glial findings to those of Öngür et al. [8], and their own findings in major depressive disorder in BA9, and contrasted them with the gliosis which would have been expected were BD a neurodegenerative disorder. Cotter et al. [36], using a two-dimensional counting method, did not replicate the findings of Rajkowska et al. [24], with no differences in neuronal density and only a trend reduction in glial density, limited to layer VI; Cotter et al. [36] did find a significant reduction in neuronal size in layers V and VI. Using the same tissue, Uranova et al. (2004) counted putative oligodendrocytes, and reported that density of these glial cells was decreased in layer VI [44]; the same group later described a reduction of perineuronal oligodendrocytes in layer III [58]. In the only study of its kind in BD, Golgi staining was used to quantify dendritic parameters of deep layer III pyramidal neurons, and a reduction in dendritic spine number and density, and dendritic length, were identified [85].

Two studies have counted sub-populations of DLPFC interneurons defined by immunoreactivity for the calcium binding proteins calbindin (CB), calretinin (CR) or PV [32, 62]. Beasley et al. [32] found a reduced density of CB-immunoreactive neurons in layers II and III, with no significant differences in CR- or PV-positive neuron. When these data were meta-analysed together with findings from a much smaller study [62], there was a reduction in BD of CB-positive neurons, significant in layers II and III, and overall if all five layers are considered together (Fig. 3). Meta-analysis of these papers also showed a reduction of PV-positive neurons across all layers, but with no significant difference in any one layer (Supplementary Figure 3); CR-positive neurons were unaffected (Supplementary Figure 4). We also meta-analysed studies of DLPFC grey matter thickness (Supplementary Figure 5) and the  density of oligodendrocytes in DLPFC white matter (Supplementary Figure 6): neither showed alterations in BD.

In summary, a range of alterations in neuronal and glial morphometry have been reported in DLPFC in BD, but apart from a decrease in density of CB-positive neurons, no specific finding has been replicated.

### Amygdala

The central role of the amygdala in arousal and affect [102, 103], its strong connections with the prefrontal cortex [5], and imaging data in BD [1, 5], has made it a structure of neuropathological interest in the disorder [5, 12, 102, 103]. Table 4 summarises the eight studies to date; three considered the amygdala as a single structure, whilst the others focused on one or more amygdaloid nuclei or groupings thereof [104].

The first study, by Bowley et al. in 2002 [34], was conducted to determine whether the amygdala shared the glial reductions seen in ACC and DLPFC. The result was negative, as were subsequent counts of glial subpopulations [43, 54, 65, 70]. Berretta et al. [53] reported a decreased volume of the lateral nucleus, which was confirmed by Pantazopoulos et al. [92] in an expanded sample, and who also described a decreased volume of the cortical nucleus. Accompanying the decreased size of the lateral nucleus, reduced neuronal size [54], and a lower neuronal number and density [53] was found. The neuronal reduction in the lateral nucleus is in part due to a loss of somatostatin (SS)-positive neurons [92], whereas in the cortical nucleus, neuropeptide Y-immunoreactive neurons were decreased [92]. The number and density of PV-immunoreactive neurons were not changed in either nucleus [70]. We were able to meta-analyse the two independent studies which measured neuronal density in lateral, basal and accessory basal nuclei [53, 54]. This revealed significant reductions in all three nuclei in BD (Fig. 4). However, this conclusion is weakened by the fact that Altshuler et al. [65], studying the same material as ref. 53 but with a different delineation of the ‘basolateral’ amygdala, found no diagnostic effect.

In summary, studies of the amygdala are consistent in showing no alterations in glia in BD, with moderate evidence for reduced density of neurons in lateral, basal and accessory basal nuclei.

### Hippocampus

Neuropathological studies of the hippocampus (including the subicular complex) in BD are summarised in Table 5.

Like PFC, the hippocampus was studied in BD in part because it had been a major focus in schizophrenia [105, 106]. Indeed, the first such study in BD, by Benes et al. [9], primarily reported schizophrenia data but also included four BD subjects, and found a reduction of neuronal density (in both disorders), selective to CA2 subfield, and affecting non-pyramidal neurons (i.e. interneurons) but not pyramidal neurons. Non-pyramidal neurons in BD were also slightly smaller. Zhang and Reynolds [39] found a markedly reduced density of PV-positive interneurons in all subfields, and a reduced size of these neurons. The next substantive report was in 2011, when Konradi and colleagues carried out a larger, stereological study to measure hippocampal volume and the number and size of neurons, including interneurons labelled by PV or SS [73]. They found a selective reduction in the volume, and the somal volume, of the non-pyramidal sector of CA2/3, The numbers of both neuropeptide-delineated interneurons were reduced in BD, across all CA fields; pyramidal neurons were unaffected. In a related study, Wang et al. [74] reported PV and SS neuron reductions in the parasubiculum. In a stereological study of the posterior hippocampus, Malchow et al. [88] found no differences in hippocampal subfield volumes in BD (including CA2/3), and an increased number and density of neurons in CA1 and in subiculum; they did not distinguish pyramidal from non-pyramidal neurons. In CA1, oligodendrocyte number was also increased. Meta-analysing the two studies of hippocampal neuron number [73, 88] revealed no differences in any subfield in BD (Supplementary Figure 7; see also Supplementary Table 5).

In summary, there is consistent albeit inconclusive evidence in the hippocampus for reductions of non-pyramidal neurons, especially of PV-positive neurons, although differences in methodology and subfields measured precluded meta-analysis. Evidence for involvement of other cell types, or for an altered hippocampal volume, is not compelling.

### Other brain regions

Seven neuropathological studies have examined the entorhinal cortex in BD (Supplementary Table 3). The only replicated positive finding is that the density of PV-immunoreactive neurons is reduced [57, 74]. An unchanged density of GFAP-positive astrocytes has been reported in two studies [28, 70], complementing a lack of alteration in overall glial density [34].

Neuropathological investigations of other neocortical regions, sometimes included to determine the anatomical selectivity of changes found in ACC or DLPFC, are essentially negative, as summarised in Supplementary Table 4. This includes a lack of alterations in the density of neurons or glia; the only exception is Brauch et al. (2007) who reported a modest increase in neuronal density in an unspecified region of temporal cortex [51].

In addition to the amygdala, several other subcortical nuclei and regions have been investigated in BD (Table 6). Various differences have been reported, particularly in the hypothalamus [18, 47, 49, 79], but no specific finding has been replicated.

## Discussion

The existence and nature of the neuropathology of psychiatric disorders has been debated for well over a century. Contemporary studies began in earnest in the 1980s, with a focus on schizophrenia, with findings in that disorder informing and encouraging equivalent investigations of other psychiatric disorders, including BD. The latter literature now extends to over 100 empirical studies, of which 81 met our criteria for inclusion in this systematic review. Our findings can be summarised as follows. First, although this is a significant body of work, most studies have relied on a limited number of brain collections, and have used relatively small sample sizes (Table 1) and are thus vulnerable to both type I and type II errors. Second, surprisingly few studies have attempted to replicate closely a previous one, precluding substantial meta-analyses; such that the latter were all limited to analysis of two studies each, comprising 16–36 BD cases and 16–74 controls (summarised in Supplementary Table 5). Hence, no neuropathological findings in BD can be considered to have been unequivocally established. Nevertheless, several findings are significant after meta-analysis of the available data, and merit brief discussion. We also consider the evidence against the presence of gliosis.

### Key positive findings

The sgACC remains of interest, with the findings of glial deficits (Fig. 1) and a thinning of grey matter (Fig. 2) being significant after meta-analysis. Indeed, the latter finding is probably the most robust positive result, given that it is based on two independent studies [26, 82], both using relatively large samples (and comprising the largest combined sample), coupled with a third study showing a similar albeit non-significant trend [78], and the absence of any contradictory reports. The results mean that further neuropathological investigations of the sgACC are warranted, especially given the continuing focus on this region for the pathophysiology and therapeutics of mood disorder [107, 108]. Whether a similar pathology is seen in other parts of the ACC is unclear, since whilst there is some evidence for a thinner layer V (Supplementary Figure 2), glial and neuronal density data are inconsistent, as discussed earlier.

The finding of a reduced density of CB-positive neurons in some layers of DLPFC is significant by meta-analysis (Fig. 3), albeit based on a modest sample size. Caution is also needed when interpreting the summary statistic which arises from considering the cortical layers together, such as the finding of reduced PV-positive neuron density (Supplementary Figure 6), especially given the lack of significant reduction of PV neurons in any one layer. On the other hand, these preliminary indications that interneurons may be affected in BD are supported by findings of a decreased density or number of interneurons, defined by a range of markers, in several brain regions [22, 23, 39, 57, 74, 92, 93]. No firm conclusions can be drawn regarding these disparate observations, but they do merit further investigation, and complement the well-established involvement of some interneuron populations in schizophrenia [109–112].

Reduced neuronal density has been identified in three nuclei of the amygdala (lateral, basal and accessory basal), arising from two reasonably sized samples by independent investigators using different methodologies ([53, 54]; Fig. 4). The findings support an involvement of the amygdala in the key circuits of BD [5, 12]. Data in other nuclei are insufficient to determine whether the findings reflect a broader distribution of amygdala changes, although the unaltered neuronal density seen in the amygdala as a whole [34] suggests that connections and functions of the laterobasal group of the amygdala may be particularly involved in BD [113–116]. However, as noted earlier, Altshuler et al. [65], using the same brains as Bezchilbnyk et al. [54], found no difference in neuronal density in BD in the basolateral nucleus. They do not define this structure, but the term conventionally refers to the lateral subdivision of the basal nucleus [104], and hence would have been subsumed within the latter region as measured in refs. 53 and 54.

### Absence of gliosis

Set against these positive findings, all of which remain to be confirmed beyond doubt, it is worth noting perhaps the clearest conclusion from this systematic review. That is, gliosis (an increase in the density, number or size of glia, especially astrocytes) is not a feature of BD. As summarised in Supplementary Table 6a, no increase in overall glial density has been reported in any of the 19 studies which have measured this parameter (and 4 of them reported reductions). Similarly, of the 12 studies which counted astrocytes (either as identified on Nissl stains, or using immunostaining), 10 reported no differences in BD, and 2 found a reduction (Supplementary Table 6b). Data for oligodendrocytes and microglia are fewer, but again show no consistent pattern of alteration (Supplementary Table 6c and 6d). Since astrocytic gliosis is usually considered to be indicative of a neurodegenerative process [117, 118], this negative profile of results suggests strongly that BD is not a disorder of that kind. Similarly, the unchanged density of microglia provides no support for the presence of an underlying neuroinflammatory process. When drawing these conclusions, it should be noted that psychiatric brain banks usually exclude subjects if formal neuropathological examination revealed specific abnormalities,  because they are viewed as coincidental and confounding findings. For example, the Stanley Foundation brain collection, used in almost half the studies included here, screened brains ‘to rule out Alzheimer’s disease and other cerebral pathology’ [10]. Nevertheless, as noted by others, the cumulative evidence is strong that BD, like other major psychiatric disorders, is not neurodegenerative in nature [7, 11, 24, 105, 117, 119]. By default, these disorders are often viewed as being neurodevelopmental in origin, although the positive evidence in favour of that conclusion comes primarily from epidemiology and functional genomics rather than from neuropathology [120–124].

### Interpreting the neuropathological findings

MRI studies show reductions in grey matter thickness in several cortical regions in BD, including ACC [2, 125]. As noted, there is also good neuropathological evidence for a thinning of sgACC (Fig. 2). However, in all other cortical areas examined, post mortem studies show minimal or no difference in grey matter thickness in BD [24–26, 32, 40, 42, 48, 66], in contrast to the anatomically widespread MRI findings. There is also a divergence between the robust MRI evidence for decreased volumes of the hippocampus [1] and most of its constituent subfields [126], and the neuropathological studies which are divided, with two reporting reduced hippocampal size [47, 73] and two which do not [9, 88]. When reconciling observations from the two modalities, it should be borne in mind that the imaging data are based on findings from over 1700 BD patients and 2500 controls, and the differences between BD and controls for each parameter are only 1–2% [1, 2]. Hence the neuropathological studies (which are about two orders of magnitude smaller; Table 1) are grossly underpowered to detect such differences. It is also possible that the group differences seen on neuroimaging are not exclusively reflective of brain structure, but have other potential interpretations and confounders [127], including the effects of lithium [128, 129].

The diagnostic status of BD and its relationships with schizophrenia and major depressive disorder continue to be under active debate clinically and genetically. This issue also has a neuropathological dimension. As noted earlier, the majority of BD studies also include one or both of these other disorders. Although it is beyond the scope of this systematic review to perform a comparative analysis, it is apparent that there is no consistent pattern of similarities or differences between these disorders (with the exception of the absence of gliosis, which is a common observation). Thus, some reported positive findings are specific to BD (e.g. [54]), some are common to all three disorders (e.g. [40, 44]), some affect BD and schizophrenia (e.g [26]), and others are shared by BD and major depressive disorder (e.g. [48]). Equally, other parameters are altered in schizophrenia and/or major depressive disorder but not in BD (e.g. [27, 63]). Overall, therefore, the neuropathological data are in line with the view, supported strongly by genomic findings [130, 131], that BD, schizophrenia and major depressive disorder are not distinct disorders, but have many features in common as well as some which distinguish them. It is also possible that some of the heterogeneity in the neuropathological data reflects the fact that there are morphological correlates of the genetic predisposition to BD as well as to the syndrome itself [132, 133].

This latter point relates to perhaps the most fundamental interpretational issue. The nature of the findings – modest reductions in volume, and in the content of neurons or glia, in certain brain regions – cannot be assumed to be pathological in the sense that lesions such as neurofibrillary tangles or infarcts are. They might instead reflect pre-existing (and partly genetically-mediated) differences in brain structure and connectivity which render the person vulnerable to BD. Or, the extant morphometric findings could be secondary to the illness in some way, e.g. cell loss or atrophy secondary to chronic stress, reduced neurotrophic factor support, etc. These issues are impossible to disentangle using post mortem studies alone, and require triangulation of neuropathological data with other findings, such as neuroimaging and relevant model systems.

### Limitations

In addition to the diagnostic issues and power considerations mentioned earlier, the literature has several other limitations to consider. The first concerns clinical phenotyping. For most studies, there is sparse information available, for example regarding the age of onset and main features of BD; the mood state at death; the presence of comorbid disorders, etc. In any event, the small sample sizes preclude any meaningful attempts at subdividing BD or correlating clinical or demographic variables with neuropathological parameters. Even the clinical diagnosis of BD itself is not straightforward when made on retrospective review of case notes or interview with relatives: Deep-Soboslay et al. [134] found that only about half of cases referred to their brain bank as BD met (DSM-IV) diagnostic criteria, with the remainder having inadequate documentation and/or substantial comorbid substance abuse. Other variables such as family history, brain hemisphere and sex could also influence neuropathological findings (e.g., refs. [8, 82, 88]), but have not been reported consistently or in sufficient detail to allow us to examine these factors. There may also be confounding by medications used in BD, since mood stabilisers, antipsychotics and antidepressants can all impact on neuronal and glial indices; such effects may either contribute to, or mitigate, the reported alterations [135–139]. Finally, the neuropathological studies of BD have been of variable methodological quality. For example, only the minority unequivocally meet stereological criteria; the remainder are subject to the limitations and potential biases of studies which do not adhere to these principles [140, 141]. Also, studies differ in the statistical approaches taken, such as whether significance values were adjusted for multiple comparisons (e.g. for the number of cortical layers examined).

## Conclusions

There remain no neuropathological correlates of BD of sufficient robustness, magnitude, and specificity, to be of clinical or diagnostic value. Clearly, this does not rule out the possibility, but it is unlikely that a neuropathology - in the conventional sense of the term - exists and which has avoided discovery. Nevertheless, the key findings of this systematic review do merit further study to either confirm or refute them. This would require research of a much larger scale and scope than has occurred to date, to ensure the results are conclusive, and to allow assessment of potential clinico-pathological correlates and subgroupings. This would be a challenging undertaking, but transcriptomic and other molecular studies of psychiatric disorders, including BD, now routinely include many hundreds of brains (e.g. refs. [123, 142, 143]). Neuropathological research should have similar aspirations.

## Electronic supplementary material

Supplementary Tables

Supplementary Figures
