# Supplementary material for: The neuropathology of bipolar disorder: systematic review and meta-analysis
Source: Mol Psychiatry. 2018 Aug 20;25(8):1787–808. doi: 10.1038/s41380-018-0213-3 (PMC6292507; doi:10.1038/s41380-018-0213-3)
Supplement: Supplementary file 1 — Supplementary Tables [file 41380_2018_213_MOESM1_ESM.docx]

**Supplementary Tables for:**

**Harrison PJ, Colbourne L, Harrison CH. The neuropathology of bipolar disorder: systematic review and meta-analysis.**

Supplementary Table 1. The 10 most cited data papers on the neuropathology of BD.

Supplementary Table 2. The 22 studies excluded from the systematic review, and the reason for exclusion.

Supplementary Table 3. Studies of entorhinal cortex.

Supplementary Table 4. Studies of temporal, parietal and occipital cortex.

Supplementary Table 5. Summary of parameters meta-analysed and the findings.

Supplementary Table 6. Summary of studies measuring glia or glial subpopulations.

**Supplementary Table 1: The ten most cited papers on the neuropathology of bipolar disorder.**

| **Year** | **Paper** | **Reference** | **Times cited** |
| --- | --- | --- | --- |
| 1998 | Öngür D, Drevets WC, Price JL. Glial reduction in the subgenual prefrontal cortex in mood disorders. *Proc Natl Acad Sci USA* 95: 13290-13295. | [8] | 973 |
| 2000 | Guidotti A, Auta J, Davis JM, DiGiorgi Gerevini V, Dwivedi Y, Grayson DR, Impagniatello F, Pandey G, Pesold C, Sharma R, Uzunov D, Costo E. Decrease in reelin and glutamic acid decarboylase_67_ (GAD_67_) expression in schizophrenia and bipolar disorder. A postmortem brain study. *Arch Gen Psychiatry* 57: 1061-1069. | [23] | 680 |
| 2001 | Cotter D, Mackay D, Landau S, Kerwin R, Everall I. Reduced glial cell density and neuronal size in the anterior cingulate cortex in major depressive disorder. *Arch Gen Psychiatry* 58: 545-553. | [27] | 448 |
| 2004 | Uranova N, Vostrikov VM, Orlovskaya DD, Rachmanova VI. Oligodendroglial density in the prefrontal cortex in schizophrenia and mood disorders: a study from the Stanley Neuropathology Consortium. *Schizophr Res* 67: 269-275. | [44] | 379 |
| 2001 | Rajkowska G, Halaris A, Selemon LD. Reductions in neuronal and glial density characterize the dorsolateral prefrontal cortex in bipolar disorder. *Biol Psychiatry* 49: 741-752. | [24] | 370 |
| 2002b | Cotter D, Mackay D, Chana G, Beasley C, Landau S, Everall IP. Reduced neuronal size and glial cell density in area 9 of the dorsolateral prefrontal cortex in subjects with major depressive disorder. *Cerebral Cortex* 12: 386-394. | [36] | 357 |
| 1998 | Benes FM, Kwok EW, Vincent SL, Todtenkopf MS. A reduction of nonpyramidal cells in sector CA2 of schizophrenics and manic depressives. *Biol Psychiatry* 44: 88-97. | [9] | 316 |
| 2001 | Uranova N, Orlovskaya D, Vikhereva O, Zimina I, Kolomeets N, Vostrikov V, Rachmanova V. Electron microscopy of oligodendroglia in severe mental illness. *Brain Res Bull* 55: 597-610. | [29] | 309 |
| 2002 | Bowley MP, Drevets WC, Öngür D, Price JL. Low glial numbers in the amygdala in major depressive disorder. *Biol Psychiatry* 52: 404-412. | [34] | 305 |
| 1996 | Purba JS, Hoogendijk WJG, Hofman MA, Swaab DF. Increased numbers of vasopressin- and oxytocin-expressing neurons in the paraventricular nucleus of the hypothalamus in depression. *Arch Gen Psychiatry* 53: 137-143. | [18] | 290 |

Citation data from Web of Science, accessed 12^th^ June 2018.

**Supplementary Table 2. The 22 excluded studies and reason for their exclusion.**

| **Paper** | **Reason**  **for exclusion^a^** |
| --- | --- |
| Raadsheer FC, Hoogendijk WJG, Stam FC, TIlders FJH, Swaab DF (1994) Increased numbers of corticotropin-releasing hormone expressing neurons in the hypothalamic paraventricular nucleus of depressed patients. *Neuroendocrinol* 60: 436-444. | 3 |
| [Bernstein HG](https://www.ncbi.nlm.nih.gov/pubmed/?term=Bernstein%20HG%5BAuthor%5D&cauthor=true&cauthor_uid=9789904), [Krell D](https://www.ncbi.nlm.nih.gov/pubmed/?term=Krell%20D%5BAuthor%5D&cauthor=true&cauthor_uid=9789904), [Baumann B](https://www.ncbi.nlm.nih.gov/pubmed/?term=Baumann%20B%5BAuthor%5D&cauthor=true&cauthor_uid=9789904), [Danos P](https://www.ncbi.nlm.nih.gov/pubmed/?term=Danos%20P%5BAuthor%5D&cauthor=true&cauthor_uid=9789904), [Falkai P](https://www.ncbi.nlm.nih.gov/pubmed/?term=Falkai%20P%5BAuthor%5D&cauthor=true&cauthor_uid=9789904), [Diekmann S](https://www.ncbi.nlm.nih.gov/pubmed/?term=Diekmann%20S%5BAuthor%5D&cauthor=true&cauthor_uid=9789904), [Henning H](https://www.ncbi.nlm.nih.gov/pubmed/?term=Henning%20H%5BAuthor%5D&cauthor=true&cauthor_uid=9789904), [Bogerts B](https://www.ncbi.nlm.nih.gov/pubmed/?term=Bogerts%20B%5BAuthor%5D&cauthor=true&cauthor_uid=9789904) (1998) Morphometric studies of the entorhinal cortex in neuropsychiatric patients and controls: clusters of heterotopically displaced lamina II neurons are not indicative of schizophrenia. [*Schizophr Res*.](https://www.ncbi.nlm.nih.gov/pubmed/9789904) 33:125-32. | 2,3 |
| [Bernstein HG](https://www.ncbi.nlm.nih.gov/pubmed/?term=Bernstein%20HG%5BAuthor%5D&cauthor=true&cauthor_uid=9483570), [Stanarius A](https://www.ncbi.nlm.nih.gov/pubmed/?term=Stanarius%20A%5BAuthor%5D&cauthor=true&cauthor_uid=9483570), [Baumann B](https://www.ncbi.nlm.nih.gov/pubmed/?term=Baumann%20B%5BAuthor%5D&cauthor=true&cauthor_uid=9483570), [Henning H](https://www.ncbi.nlm.nih.gov/pubmed/?term=Henning%20H%5BAuthor%5D&cauthor=true&cauthor_uid=9483570), [Krell D](https://www.ncbi.nlm.nih.gov/pubmed/?term=Krell%20D%5BAuthor%5D&cauthor=true&cauthor_uid=9483570), [Danos P](https://www.ncbi.nlm.nih.gov/pubmed/?term=Danos%20P%5BAuthor%5D&cauthor=true&cauthor_uid=9483570), [Falkai P](https://www.ncbi.nlm.nih.gov/pubmed/?term=Falkai%20P%5BAuthor%5D&cauthor=true&cauthor_uid=9483570), [Bogerts B](https://www.ncbi.nlm.nih.gov/pubmed/?term=Bogerts%20B%5BAuthor%5D&cauthor=true&cauthor_uid=9483570) (1998) Nitric oxide synthase-containing neurons in the human hypothalamus: reduced number of immunoreactive cells in the paraventricular nucleus of depressive patients and schizophrenics. [*Neuroscience*.](https://www.ncbi.nlm.nih.gov/pubmed/9483570) 83: 867-75. | 2 |
| [Baumann B](https://www.ncbi.nlm.nih.gov/pubmed/?term=Baumann%20B%5BAuthor%5D&cauthor=true&cauthor_uid=9990559), [Danos P](https://www.ncbi.nlm.nih.gov/pubmed/?term=Danos%20P%5BAuthor%5D&cauthor=true&cauthor_uid=9990559), [Krell D](https://www.ncbi.nlm.nih.gov/pubmed/?term=Krell%20D%5BAuthor%5D&cauthor=true&cauthor_uid=9990559), [Diekmann S](https://www.ncbi.nlm.nih.gov/pubmed/?term=Diekmann%20S%5BAuthor%5D&cauthor=true&cauthor_uid=9990559), [Leschinger A](https://www.ncbi.nlm.nih.gov/pubmed/?term=Leschinger%20A%5BAuthor%5D&cauthor=true&cauthor_uid=9990559), [Stauch R](https://www.ncbi.nlm.nih.gov/pubmed/?term=Stauch%20R%5BAuthor%5D&cauthor=true&cauthor_uid=9990559), [Wurthmann C](https://www.ncbi.nlm.nih.gov/pubmed/?term=Wurthmann%20C%5BAuthor%5D&cauthor=true&cauthor_uid=9990559), [Bernstein HG](https://www.ncbi.nlm.nih.gov/pubmed/?term=Bernstein%20HG%5BAuthor%5D&cauthor=true&cauthor_uid=9990559), [Bogerts B](https://www.ncbi.nlm.nih.gov/pubmed/?term=Bogerts%20B%5BAuthor%5D&cauthor=true&cauthor_uid=9990559) (1999) Reduced volume of limbic system-affiliated basal ganglia in mood disorders: preliminary data from a postmortem study. [*J Neuropsychiatry Clin Neurosci*.](https://www.ncbi.nlm.nih.gov/pubmed/9990559) 11: 71-8. | 2 |
| [Bayer TA](https://www.ncbi.nlm.nih.gov/pubmed/?term=Bayer%20TA%5BAuthor%5D&cauthor=true&cauthor_uid=10477118), [Buslei R](https://www.ncbi.nlm.nih.gov/pubmed/?term=Buslei%20R%5BAuthor%5D&cauthor=true&cauthor_uid=10477118), [Havas L](https://www.ncbi.nlm.nih.gov/pubmed/?term=Havas%20L%5BAuthor%5D&cauthor=true&cauthor_uid=10477118), [Falkai P](https://www.ncbi.nlm.nih.gov/pubmed/?term=Falkai%20P%5BAuthor%5D&cauthor=true&cauthor_uid=10477118) (1999) Evidence for activation of microglia in patients with psychiatric illnesses. [*Neurosci Lett*.](https://www.ncbi.nlm.nih.gov/pubmed/10477118) 271: 126-8. | 2,3 |
| [Benes FM](https://www.ncbi.nlm.nih.gov/pubmed/?term=Benes%20FM%5BAuthor%5D&cauthor=true&cauthor_uid=11207424), [Todtenkopf MS](https://www.ncbi.nlm.nih.gov/pubmed/?term=Todtenkopf%20MS%5BAuthor%5D&cauthor=true&cauthor_uid=11207424), [Logiotatos P](https://www.ncbi.nlm.nih.gov/pubmed/?term=Logiotatos%20P%5BAuthor%5D&cauthor=true&cauthor_uid=11207424), [Williams M](https://www.ncbi.nlm.nih.gov/pubmed/?term=Williams%20M%5BAuthor%5D&cauthor=true&cauthor_uid=11207424) (2000) Glutamate decarboxylase(65)-immunoreactive terminals in cingulate and prefrontal cortices of schizophrenic and bipolar brain. [*J Chem Neuroanat*.](https://www.ncbi.nlm.nih.gov/pubmed/11207424) 20: 259-69. | 4 |
| [Rosoklija G](https://www.ncbi.nlm.nih.gov/pubmed/?term=Rosoklija%20G%5BAuthor%5D&cauthor=true&cauthor_uid=10768696), [Toomayan G](https://www.ncbi.nlm.nih.gov/pubmed/?term=Toomayan%20G%5BAuthor%5D&cauthor=true&cauthor_uid=10768696), [Ellis SP](https://www.ncbi.nlm.nih.gov/pubmed/?term=Ellis%20SP%5BAuthor%5D&cauthor=true&cauthor_uid=10768696), [Keilp J](https://www.ncbi.nlm.nih.gov/pubmed/?term=Keilp%20J%5BAuthor%5D&cauthor=true&cauthor_uid=10768696), [Mann JJ](https://www.ncbi.nlm.nih.gov/pubmed/?term=Mann%20JJ%5BAuthor%5D&cauthor=true&cauthor_uid=10768696), [Latov N](https://www.ncbi.nlm.nih.gov/pubmed/?term=Latov%20N%5BAuthor%5D&cauthor=true&cauthor_uid=10768696), [Hays AP](https://www.ncbi.nlm.nih.gov/pubmed/?term=Hays%20AP%5BAuthor%5D&cauthor=true&cauthor_uid=10768696), [Dwork AJ](https://www.ncbi.nlm.nih.gov/pubmed/?term=Dwork%20AJ%5BAuthor%5D&cauthor=true&cauthor_uid=10768696) (2000) Structural abnormalities of subicular dendrites in subjects with schizophrenia and mood disorders: preliminary findings. [*Arch Gen Psychiatry*.](https://www.ncbi.nlm.nih.gov/pubmed/10768696) 57: 349-56. | 3 |
| [Dowlatshahi D](https://www.ncbi.nlm.nih.gov/pubmed/?term=Dowlatshahi%20D%5BAuthor%5D&cauthor=true&cauthor_uid=11117489), [MacQueen G](https://www.ncbi.nlm.nih.gov/pubmed/?term=MacQueen%20G%5BAuthor%5D&cauthor=true&cauthor_uid=11117489), [Wang JF](https://www.ncbi.nlm.nih.gov/pubmed/?term=Wang%20JF%5BAuthor%5D&cauthor=true&cauthor_uid=11117489), [Chen B](https://www.ncbi.nlm.nih.gov/pubmed/?term=Chen%20B%5BAuthor%5D&cauthor=true&cauthor_uid=11117489), [Young LT](https://www.ncbi.nlm.nih.gov/pubmed/?term=Young%20LT%5BAuthor%5D&cauthor=true&cauthor_uid=11117489) (2000) Increased hippocampal supragranular Timm staining in subjects with bipolar disorder. [*Neuroreport*.](https://www.ncbi.nlm.nih.gov/pubmed/11117489) 11: 3775-8. | 4 |
| [Zhou JN](https://www.ncbi.nlm.nih.gov/pubmed/?term=Zhou%20JN%5BAuthor%5D&cauthor=true&cauthor_uid=11448372), [Riemersma RF](https://www.ncbi.nlm.nih.gov/pubmed/?term=Riemersma%20RF%5BAuthor%5D&cauthor=true&cauthor_uid=11448372), [Unmehopa UA](https://www.ncbi.nlm.nih.gov/pubmed/?term=Unmehopa%20UA%5BAuthor%5D&cauthor=true&cauthor_uid=11448372), [Hoogendijk WJ](https://www.ncbi.nlm.nih.gov/pubmed/?term=Hoogendijk%20WJ%5BAuthor%5D&cauthor=true&cauthor_uid=11448372), [van Heerikhuize JJ](https://www.ncbi.nlm.nih.gov/pubmed/?term=van%20Heerikhuize%20JJ%5BAuthor%5D&cauthor=true&cauthor_uid=11448372), [Hofman MA](https://www.ncbi.nlm.nih.gov/pubmed/?term=Hofman%20MA%5BAuthor%5D&cauthor=true&cauthor_uid=11448372), [Swaab DF](https://www.ncbi.nlm.nih.gov/pubmed/?term=Swaab%20DF%5BAuthor%5D&cauthor=true&cauthor_uid=11448372) (2001) Alterations in arginine vasopressin neurons in the suprachiasmatic nucleus in depression. [*Arch Gen Psychiatry*.](https://www.ncbi.nlm.nih.gov/pubmed/11448372) 58: 655-62. | 3 |
| [Chambers JS](https://www.ncbi.nlm.nih.gov/pubmed/?term=Chambers%20JS%5BAuthor%5D&cauthor=true&cauthor_uid=15672553), [Perrone-Bizzozero NI](https://www.ncbi.nlm.nih.gov/pubmed/?term=Perrone-Bizzozero%20NI%5BAuthor%5D&cauthor=true&cauthor_uid=15672553) (2004) Altered myelination of the hippocampal formation in subjects with schizophrenia and bipolar disorder. [*Neurochem Res*.](https://www.ncbi.nlm.nih.gov/pubmed/15672553) 29: 2293-302. | 1 |
| [Dorph-Petersen KA](https://www.ncbi.nlm.nih.gov/pubmed/?term=Dorph-Petersen%20KA%5BAuthor%5D&cauthor=true&cauthor_uid=15065119), [Pierri JN](https://www.ncbi.nlm.nih.gov/pubmed/?term=Pierri%20JN%5BAuthor%5D&cauthor=true&cauthor_uid=15065119), [Sun Z](https://www.ncbi.nlm.nih.gov/pubmed/?term=Sun%20Z%5BAuthor%5D&cauthor=true&cauthor_uid=15065119), [Sampson AR](https://www.ncbi.nlm.nih.gov/pubmed/?term=Sampson%20AR%5BAuthor%5D&cauthor=true&cauthor_uid=15065119), [Lewis DA](https://www.ncbi.nlm.nih.gov/pubmed/?term=Lewis%20DA%5BAuthor%5D&cauthor=true&cauthor_uid=15065119) (2004) Stereological analysis of the mediodorsal thalamic nucleus in schizophrenia: volume, neuron number, and cell types. [*J Comp Neurol*.](https://www.ncbi.nlm.nih.gov/pubmed/15065119) 472: 449-62. | 2 |
| [Dorph-Petersen KA](https://www.ncbi.nlm.nih.gov/pubmed/?term=Dorph-Petersen%20KA%5BAuthor%5D&cauthor=true&cauthor_uid=18642008), [Caric D](https://www.ncbi.nlm.nih.gov/pubmed/?term=Caric%20D%5BAuthor%5D&cauthor=true&cauthor_uid=18642008), [Saghafi R](https://www.ncbi.nlm.nih.gov/pubmed/?term=Saghafi%20R%5BAuthor%5D&cauthor=true&cauthor_uid=18642008), [Zhang W](https://www.ncbi.nlm.nih.gov/pubmed/?term=Zhang%20W%5BAuthor%5D&cauthor=true&cauthor_uid=18642008), [Sampson AR](https://www.ncbi.nlm.nih.gov/pubmed/?term=Sampson%20AR%5BAuthor%5D&cauthor=true&cauthor_uid=18642008), [Lewis DA](https://www.ncbi.nlm.nih.gov/pubmed/?term=Lewis%20DA%5BAuthor%5D&cauthor=true&cauthor_uid=18642008) (2009) Volume and neuron number of the lateral geniculate nucleus in schizophrenia and mood disorders. [*Acta Neuropathol*.](https://www.ncbi.nlm.nih.gov/pubmed/18642008) 117: 369-84. | 2 |
| [Jellinger KA](https://www.ncbi.nlm.nih.gov/pubmed/?term=Jellinger%20KA%5BAuthor%5D&cauthor=true&cauthor_uid=19198857) (2009) Lewy body/alpha-synucleinopathy in schizophrenia and depression: a preliminary neuropathological study. [*Acta Neuropathol.*](https://www.ncbi.nlm.nih.gov/pubmed/19198857) 117: 423-7. | 3 |
| [Gittins RA](https://www.ncbi.nlm.nih.gov/pubmed/?term=Gittins%20RA%5BAuthor%5D&cauthor=true&cauthor_uid=21497910), [Harrison PJ](https://www.ncbi.nlm.nih.gov/pubmed/?term=Harrison%20PJ%5BAuthor%5D&cauthor=true&cauthor_uid=21497910) (2011) A morphometric study of glia and neurons in the anterior cingulate cortex in mood disorder. [*J Affect Disord*.](https://www.ncbi.nlm.nih.gov/pubmed/21497910) 133: 328-32. | 2 |
| [Hayashi](https://www.ncbi.nlm.nih.gov/pubmed/?term=Hayashi%20Y%5BAuthor%5D&cauthor=true&cauthor_uid=22438888) Y, [Nihonmatsu-Kikuchi](https://www.ncbi.nlm.nih.gov/pubmed/?term=Nihonmatsu-Kikuchi%20N%5BAuthor%5D&cauthor=true&cauthor_uid=22438888) N, [Hisanaga](https://www.ncbi.nlm.nih.gov/pubmed/?term=Hisanaga%20Si%5BAuthor%5D&cauthor=true&cauthor_uid=22438888) S, [Yu](https://www.ncbi.nlm.nih.gov/pubmed/?term=Yu%20Xj%5BAuthor%5D&cauthor=true&cauthor_uid=22438888) X, and [Tatebayashi](https://www.ncbi.nlm.nih.gov/pubmed/?term=Tatebayashi%20Y%5BAuthor%5D&cauthor=true&cauthor_uid=22438888) Y (2012) Neuropathological similarities and differences between Schizophrenia and bipolar disorder: A flow cytometric postmortem brain study**.** [*PLoS One*](https://www.ncbi.nlm.nih.gov/pmc/articles/PMC3305297/). 7: e33019. | 4 |
| [Oh DH](https://www.ncbi.nlm.nih.gov/pubmed/?term=Oh%20DH%5BAuthor%5D&cauthor=true&cauthor_uid=21962915), [Son H](https://www.ncbi.nlm.nih.gov/pubmed/?term=Son%20H%5BAuthor%5D&cauthor=true&cauthor_uid=21962915), [Hwang S](https://www.ncbi.nlm.nih.gov/pubmed/?term=Hwang%20S%5BAuthor%5D&cauthor=true&cauthor_uid=21962915), [Kim SH](https://www.ncbi.nlm.nih.gov/pubmed/?term=Kim%20SH%5BAuthor%5D&cauthor=true&cauthor_uid=21962915) (2012) Neuropathological abnormalities of astrocytes, GABAergic neurons, and pyramidal neurons in the dorsolateral prefrontal cortices of patients with major depressive disorder. [*Eur Neuropsychopharmacol.*](https://www.ncbi.nlm.nih.gov/pubmed/21962915) 22: 330-8. | 1 |
| [Bernstein HG](https://www.ncbi.nlm.nih.gov/pubmed/?term=Bernstein%20HG%5BAuthor%5D&cauthor=true&cauthor_uid=23462372), [Dobrowolny H](https://www.ncbi.nlm.nih.gov/pubmed/?term=Dobrowolny%20H%5BAuthor%5D&cauthor=true&cauthor_uid=23462372), [Schott BH](https://www.ncbi.nlm.nih.gov/pubmed/?term=Schott%20BH%5BAuthor%5D&cauthor=true&cauthor_uid=23462372), [Gorny X](https://www.ncbi.nlm.nih.gov/pubmed/?term=Gorny%20X%5BAuthor%5D&cauthor=true&cauthor_uid=23462372), [Becker V](https://www.ncbi.nlm.nih.gov/pubmed/?term=Becker%20V%5BAuthor%5D&cauthor=true&cauthor_uid=23462372), [Steiner J](https://www.ncbi.nlm.nih.gov/pubmed/?term=Steiner%20J%5BAuthor%5D&cauthor=true&cauthor_uid=23462372), [Seidenbecher CI](https://www.ncbi.nlm.nih.gov/pubmed/?term=Seidenbecher%20CI%5BAuthor%5D&cauthor=true&cauthor_uid=23462372), [Bogerts B](https://www.ncbi.nlm.nih.gov/pubmed/?term=Bogerts%20B%5BAuthor%5D&cauthor=true&cauthor_uid=23462372) (2013) Increased density of AKAP5-expressing neurons in the anterior cingulate cortex of subjects with bipolar disorder. [*J Psychiatr Res*.](https://www.ncbi.nlm.nih.gov/pubmed/23462372) 47: 699-705. | 4 |
| [Bielau H](https://www.ncbi.nlm.nih.gov/pubmed/?term=Bielau%20H%5BAuthor%5D&cauthor=true&cauthor_uid=24040806), [Brisch R](https://www.ncbi.nlm.nih.gov/pubmed/?term=Brisch%20R%5BAuthor%5D&cauthor=true&cauthor_uid=24040806), [Gos T](https://www.ncbi.nlm.nih.gov/pubmed/?term=Gos%20T%5BAuthor%5D&cauthor=true&cauthor_uid=24040806), [Dobrowolny H](https://www.ncbi.nlm.nih.gov/pubmed/?term=Dobrowolny%20H%5BAuthor%5D&cauthor=true&cauthor_uid=24040806), [Baumann B](https://www.ncbi.nlm.nih.gov/pubmed/?term=Baumann%20B%5BAuthor%5D&cauthor=true&cauthor_uid=24040806), [Mawrin C](https://www.ncbi.nlm.nih.gov/pubmed/?term=Mawrin%20C%5BAuthor%5D&cauthor=true&cauthor_uid=24040806), [Kreutzmann P](https://www.ncbi.nlm.nih.gov/pubmed/?term=Kreutzmann%20P%5BAuthor%5D&cauthor=true&cauthor_uid=24040806), [Bernstein HG](https://www.ncbi.nlm.nih.gov/pubmed/?term=Bernstein%20HG%5BAuthor%5D&cauthor=true&cauthor_uid=24040806), [Bogerts B](https://www.ncbi.nlm.nih.gov/pubmed/?term=Bogerts%20B%5BAuthor%5D&cauthor=true&cauthor_uid=24040806), [Steiner J](https://www.ncbi.nlm.nih.gov/pubmed/?term=Steiner%20J%5BAuthor%5D&cauthor=true&cauthor_uid=24040806) (2013) Volumetric analysis of the hypothalamus, amygdala and hippocampus in non-suicidal and suicidal mood disorder patients--a post-mortem study. [*CNS Neurol Disord Drug Targets*.](https://www.ncbi.nlm.nih.gov/pubmed/24040806) 12: 914-20. | 3 |
| [Mauney SA](https://www.ncbi.nlm.nih.gov/pubmed/?term=Mauney%20SA%5BAuthor%5D&cauthor=true&cauthor_uid=23790226), [Athanas KM](https://www.ncbi.nlm.nih.gov/pubmed/?term=Athanas%20KM%5BAuthor%5D&cauthor=true&cauthor_uid=23790226), [Pantazopoulos H](https://www.ncbi.nlm.nih.gov/pubmed/?term=Pantazopoulos%20H%5BAuthor%5D&cauthor=true&cauthor_uid=23790226), [Shaskan N](https://www.ncbi.nlm.nih.gov/pubmed/?term=Shaskan%20N%5BAuthor%5D&cauthor=true&cauthor_uid=23790226), [Passeri E](https://www.ncbi.nlm.nih.gov/pubmed/?term=Passeri%20E%5BAuthor%5D&cauthor=true&cauthor_uid=23790226), [Berretta S](https://www.ncbi.nlm.nih.gov/pubmed/?term=Berretta%20S%5BAuthor%5D&cauthor=true&cauthor_uid=23790226), [Woo TU](https://www.ncbi.nlm.nih.gov/pubmed/?term=Woo%20TU%5BAuthor%5D&cauthor=true&cauthor_uid=23790226) (2013) Developmental pattern of perineuronal nets in the human prefrontal cortex and their deficit in schizophrenia. [*Biol Psychiatry*.](https://www.ncbi.nlm.nih.gov/pubmed/23790226) 74: 427-35. | 4 |
| [Brisch R](https://www.ncbi.nlm.nih.gov/pubmed/?term=Brisch%20R%5BAuthor%5D&cauthor=true&cauthor_uid=26578879), [Bielau H](https://www.ncbi.nlm.nih.gov/pubmed/?term=Bielau%20H%5BAuthor%5D&cauthor=true&cauthor_uid=26578879), [Saniotis A](https://www.ncbi.nlm.nih.gov/pubmed/?term=Saniotis%20A%5BAuthor%5D&cauthor=true&cauthor_uid=26578879), [Wolf R](https://www.ncbi.nlm.nih.gov/pubmed/?term=Wolf%20R%5BAuthor%5D&cauthor=true&cauthor_uid=26578879), [Bogerts B](https://www.ncbi.nlm.nih.gov/pubmed/?term=Bogerts%20B%5BAuthor%5D&cauthor=true&cauthor_uid=26578879), [Krell D](https://www.ncbi.nlm.nih.gov/pubmed/?term=Krell%20D%5BAuthor%5D&cauthor=true&cauthor_uid=26578879), [Steiner J](https://www.ncbi.nlm.nih.gov/pubmed/?term=Steiner%20J%5BAuthor%5D&cauthor=true&cauthor_uid=26578879), [Braun K](https://www.ncbi.nlm.nih.gov/pubmed/?term=Braun%20K%5BAuthor%5D&cauthor=true&cauthor_uid=26578879), [Krzyżanowska M](https://www.ncbi.nlm.nih.gov/pubmed/?term=Krzy%C5%BCanowska%20M%5BAuthor%5D&cauthor=true&cauthor_uid=26578879), [Krzyżanowski M](https://www.ncbi.nlm.nih.gov/pubmed/?term=Krzy%C5%BCanowski%20M%5BAuthor%5D&cauthor=true&cauthor_uid=26578879), [Jankowski Z](https://www.ncbi.nlm.nih.gov/pubmed/?term=Jankowski%20Z%5BAuthor%5D&cauthor=true&cauthor_uid=26578879), [Kaliszan M](https://www.ncbi.nlm.nih.gov/pubmed/?term=Kaliszan%20M%5BAuthor%5D&cauthor=true&cauthor_uid=26578879), [Bernstein HG](https://www.ncbi.nlm.nih.gov/pubmed/?term=Bernstein%20HG%5BAuthor%5D&cauthor=true&cauthor_uid=26578879), [Gos T](https://www.ncbi.nlm.nih.gov/pubmed/?term=Gos%20T%5BAuthor%5D&cauthor=true&cauthor_uid=26578879) (2015) Calretinin and parvalbumin in schizophrenia and affective disorders: a mini-review, a perspective on the evolutionary role of calretinin in schizophrenia, and a preliminary post-mortem study of calretinin in the septal nuclei. [*Front Cell Neurosci*.](https://www.ncbi.nlm.nih.gov/pubmed/26578879) 9: 393. | 3 |
| [Lake EMR](https://www.ncbi.nlm.nih.gov/pubmed/?term=Lake%20EMR%5BAuthor%5D&cauthor=true&cauthor_uid=27629158), [Steffler EA](https://www.ncbi.nlm.nih.gov/pubmed/?term=Steffler%20EA%5BAuthor%5D&cauthor=true&cauthor_uid=27629158), [Rowley CD](https://www.ncbi.nlm.nih.gov/pubmed/?term=Rowley%20CD%5BAuthor%5D&cauthor=true&cauthor_uid=27629158), [Sehmbi M](https://www.ncbi.nlm.nih.gov/pubmed/?term=Sehmbi%20M%5BAuthor%5D&cauthor=true&cauthor_uid=27629158), [Minuzzi L](https://www.ncbi.nlm.nih.gov/pubmed/?term=Minuzzi%20L%5BAuthor%5D&cauthor=true&cauthor_uid=27629158), [Frey BN](https://www.ncbi.nlm.nih.gov/pubmed/?term=Frey%20BN%5BAuthor%5D&cauthor=true&cauthor_uid=27629158), [Bock NA](https://www.ncbi.nlm.nih.gov/pubmed/?term=Bock%20NA%5BAuthor%5D&cauthor=true&cauthor_uid=27629158) (2017) Altered intracortical myelin staining in the dorsolateral prefrontal cortex in severe mental illness. [*Eur Arch Psychiatry Clin Neurosci.*](https://www.ncbi.nlm.nih.gov/pubmed/27629158) 267: 369-376. | 4 |
| [Abbass M](https://www.ncbi.nlm.nih.gov/pubmed/?term=Abbass%20M%5BAuthor%5D&cauthor=true&cauthor_uid=29126813), [Trought K](https://www.ncbi.nlm.nih.gov/pubmed/?term=Trought%20K%5BAuthor%5D&cauthor=true&cauthor_uid=29126813), [Long D](https://www.ncbi.nlm.nih.gov/pubmed/?term=Long%20D%5BAuthor%5D&cauthor=true&cauthor_uid=29126813), [Semechko A](https://www.ncbi.nlm.nih.gov/pubmed/?term=Semechko%20A%5BAuthor%5D&cauthor=true&cauthor_uid=29126813), [Wong AHC](https://www.ncbi.nlm.nih.gov/pubmed/?term=Wong%20AHC%5BAuthor%5D&cauthor=true&cauthor_uid=29126813) (2018) Automated immunohistochemical method to analyze large areas of the human cortex. [*J Neurosci Methods*.](https://www.ncbi.nlm.nih.gov/pubmed/29126813) 294: 81-90. | 4 |

^a^1: No original data presented. 2: Less than 3 BD subjects. 3: BD data not separable from other diagnostic group(s). 4: No data meeting our neuropathological criteria (see Materials and Methods).

**Supplementary Table 3: Studies of entorhinal cortex**

| **Study** | **Sample size**  **(Con/BD)** | **Sub-regions investigated** | **Stain** | **Parameters measured** | **Key findings in BD** |
| --- | --- | --- | --- | --- | --- |
| Beckmann & Jakob (1991) [17] | 4/4 | Rostral; layers II, III, IV | Nissl | Features and clustering of neurons | ‘Definite cytoarchitectural abnormalities’, e.g. absence of normal pre-alpha cell islands; heterotopic neurons. No quantitative data. |
| Damazdic et al (2001) [28] | 15/13 | Intermediate; layers II-VI | GFAP | GFAP+ cell density | No differences |
| Bowley et al (2002) [34] | 10/12 | Rostral | Nissl | Density of neurons and glia | No differences |
| Damazdic et al (2002) [37] | 11/8 | Intermediate; layers II-VI | Silver | Density of neurofibrillary tangles and senile plaques | No differences |
| Pantazopoulos et al (2007) [57] | 16/10 | Whole; rostral, lateral, intermediate, caudal | Nissl, PV | Volume. Number, density and size of PV+ neurons. | Entorhinal volume unchanged. PV+ neuron number (-22%) and density (-38%) decreased overall, and in superficial layers of lateral (-22%), intermediate (-42%) and caudal (-22%) regions. PV+ somal size unchanged. |
| Pantazopoulos et al (2010) [70] | 15/11 | Rostral, lateral, intermediate, caudal | GFAP | GFAP+ cell density | No differences |
| Wang et al (2011) [74] | 17/13 | Caudal; superficial and deep layers | PV, SS, CB | Neuronal density | PV+ and SS+ neuron densities reduced. CB+ neuron density unaltered. |

CB: calbindin. GFAP: glial fibrillary acidic protein. PV: parvalbumin. SS: somatostatin.

**Supplementary Table 4: Studies of temporal, parietal and occipital cortex**

| **Study** | **Sample size**  **(Con/BD)** | **Cortical region investigated** | **Stain** | **Parameters measured** | **Key findings in BD** |
| --- | --- | --- | --- | --- | --- |
| Elvidge et al (1938) [15] | ?/5 | Occipito-parietal (biopsy) | Silver | Oligodendrocyte and astrocyte morphology | Swelling of oligodendrocytes. No quantitative data. |
| Öngür et al (1998) [8] | 13/15 | Parietal (BA3b) | Nissl | Volume; neuronal and glial density and number | No differences |
| Bouras et al (2001) [26] | 55/21 | Occipital cortex (BA18) | Nissl | Cortical thickness, neuronal density, neuronal size | No differences (data not presented) |
| Cotter et al (2004) [42] | 15/15 | Superior temporal (BA41 [Heschl’s gyrus]), layers III and V | Nissl | Cortical thickness; neuronal and glial density | No differences |
| Beasley et al (2005) [46] | 15/15 | Anterior superior temporal (planum temporale) | Nissl | Neuronal and glial density, size, and clustering | Reduced neuronal clustering. No differences in glial density. |
| Brauch et al (2007) [51] | 14/14 | Temporal | Nissl | Neuronal and glial density; area occupied by neurons and glia | Neuronal density increased (+17%). Glial density unchanged; area occupied by glia reduced (-22%). |
| Bielau et al (2007) [55] | 19/11 | Superior temporal | GAD | Neuronal density | No differences |
| Pennington et al (2008) [61] | 15/15 | Dorsocaudal insular (BA 13-16), layers II and III | Nissl | Neuronal and glial density and size | No differences |
| Beasley et al (2009) [63] | 15/15 | White matter below anterior superior temporal (planum temporale) | Nissl | Neuronal and glial density, size, and distribution | No differences |
| Bernstein et al (2015) [87] | 16/15 | Posterior cingulate (BA32) and insular cortex (BA14) | GS | Density of GS-positive astrocytes and GS-positive oligodendrocytes | No differences |

GAD: glutamic acid decarboxylase. GS: glutamine synthetase

**Supplementary Table 5. Summary of meta-analyses and the findings**

| *Region* | *Parameters meta-analysed* | *Studies included* | *Con-trols (n)* | *BD cases (n)* | *Meta-analytic finding in BD vs controls^a^* | *Data shown in* | *Notes* |
| --- | --- | --- | --- | --- | --- | --- | --- |
| sgACC | Volume; glial density and number; neuronal density and number | Öngür et al 1998 [8] combining pilot data with main study data | 16 | 18 | Reduced glial number and glial density | Fig. 1 |  |
| sgACC | Thickness of layers 2,3,5,6 | Bouras et al 2001 [26];  Williams et al 2013a [82] | 74 | 36 | Thinner layers 3,5 and 6 | Fig. 2 | Data for men and women in Williams et al 2013a [82] were combined (see Methods and Materials). Ref. 78 also measured layer III and V thickness in sgACC but presented data as medians and IQRs. |
| ACC | Thickness of layers 2,3,5,6 | Benes et al 2001 [25]; Bouras et al 2001 [26] | 67 | 31 | Thinner layer 5 | Suppl. Fig. 2 |  |
| DPFC | Density of CB, CR and PV neurons in layers 1,2,3,4,5/6 | Beasley et al 2002a [32];  Sakai et al 2008 [62] | 20 | 20 | CB neuron density reduced in layers 2 and 3, and overall. CR: no differences. PV: no difference in any layer, but overall reduction. | Fig. 3 and Suppl. Figs. 3 and 4 | Data for the ‘medium’ size class of each neuron population were used from [78]. |
| DPFC | Thickness | Rajkowska et al 2001 [24];  Beasley et al 2002a [32] | 26 | 25 | No differences | Suppl. Fig. 5 |  |
| DPFC WM | Oligodendrocyte density | Uranova et al 2004 [44]; Mosebach et al 2013 [81] | 31 | 23 | No differences | Suppl. Fig. 6 | Data averaged between right and left hemisphere for Mosebach et al 2013 [81]. |
| Amygdala | Neuronal density in lateral, basal, and accessory basal nuclei | Berretta et al 2007 [53]; Bezchilbnyk et al 2007 [54] | 27 | 21 | Reduced neuron density in all three nuclei | Fig. 4 | [53] used 3D counting, [54] used 2D counting. [54] found a neuron size difference in lateral nucleus in BD, which may confound neuronal density data. Data in [54] for accessory basal nucleus were averaged between parvocellular and magnocellular subnuclei. |
| HC | Neuron number in CA1, CA2/3, CA4 | Konradi et al 2011 [73]; Malchow et al 2015 [88] | 28 | 22 | No differences in any subfield, but overall reduction^b^ | Suppl. Fig. 7 | Data averaged between right and left hemisphere for Malchow et al 2015 [88]. |

Abbreviations: 2D: two-dimensional counting (i.e. cells per unit area). 3D: three-dimensional counting (i.e. cells per unit volume). ACCsg: subgenual ACC. ACC: non-subgenual regions of ACC. CB: calbindin. CR: calretinin. DPFC: dorsolateral prefrontal cortex. HC: hippocampus. IQR: inter-quartile range. PV: parvalbumin. WM: white matter.

^a^’Overall reduction’ refers to a significant P value for a summary statistic in which all layers or subfields were considered together. As noted in the text, this is based on N of observations not N of subjects (e.g. if 5 layers are measured in 20 subjects, N=100) and hence should be interpreted with caution.

^b^This overall reduction is not meaningful since findings in the two studies went in opposing directions in each subfield, resulting in significant heterogeneity (I^2^=86%). See Suppl. Fig. 7.

**Supplementary Table 6. Summary of studies reporting density of glia or glial subpopulations in BD.**

1. **Overall glial density, measured using Nissl staining**

| *Reduced* | *Unchanged* | *Increased* |
| --- | --- | --- |
| Öngür et al 1998 [8] (ACC) | Nasrallah et al 1983 [16] (CC) |  |
| Rajkowska et al 2001 [24] (DPFC) | Öngür et al 1998 [8] (BA3) |  |
| Cotter et al 2002b [36] (DPFC) | Benes et al 2001 [25] (ACC) |  |
| Todtenkopf et al 2005 [50] (ACC) | Cotter et al 2001 [27] (ACC) |  |
|  | Bowley et al 2002 [34] (Amygdala, ERC) |  |
|  | Chana et al 2003 [40] (ACC) |  |
|  | Cotter et al 2004 [42] (TC) |  |
|  | Hamidi et al 2004 [43] (Amygdala) |  |
|  | Beasley et al 2005 [46] (PT) |  |
|  | Cotter et al 2005 [48] (OFC) |  |
|  | Sakai et al 2008 [62] (DPFC) |  |
|  | Bezchilbnyk et al 2007 [54] (Amygdala) |  |
|  | Brauch et al 2007 [51] (TC) |  |
|  | Pennington et al 2008 [61] (Insula) |  |
|  | Beasley et al 2009 [63] (PT WM) |  |

1. **Astrocyte density, measured using Nissl, GFAP or GS staining**

| *Reduced* | *Unchanged* | *Increased* |
| --- | --- | --- |
| Toro et al 2006 [52] (OFC)^a^ | Damazdic et al 2001 [28] (ERC) |  |
| Hercher et al 2014 [84] (DPFC) | Webster et al 2001 [30] (DPFC, HC) |  |
|  | Toro et al 2006 [52] (OFC, DPFC)^b^ |  |
|  | Altshuler et al 2010 [65] (Amygdala) |  |
|  | Pantazopoulos et al 2010 [70] (Amygdala, ERC) |  |
|  | Gos et al 2013 [80] (HC) |  |
|  | Williams et al 2013b [83] (ACC,CC) |  |
|  | Williams et al 2014 [86] (ACC) |  |
|  | Bernstein et al 2015 [87] (Cortical areas, N.acc.) |  |
|  | Malchow et al 2015 [88] (HC) |  |

1. **Oligodendrocyte density, measured using Nissl or olig1 staining**

| *Reduced* | *Unchanged* | *Increased* |
| --- | --- | --- |
| Uranova et al 2004 [44] (DPFC) | Uranova et al 2004 [44] (DPFC WM) | Hercher et al 2014 [84] (DPFC) |
| Vostrikov et al 2007 [58] (DPFC) | Mosebach et al 2013 [81] (ACC, DPFC) | Malchow et al 2015 [88] (HC) |
|  | Williams et al 2013b [83] (ACC, CC) |  |
|  | Bernstein et al 2015 [87] (Cortical areas, N. acc)^b^ |  |

1. **Microglial density, measured using HLA or Iba-1 staining**

| *Reduced* | *Unchanged* | *Increased* |
| --- | --- | --- |
|  | Hamidi et al 2004 [43] (amygdala) |  |
|  | Connor et al 2009 [64] (ACC WM) |  |
|  | Hercher et al 2014 [84] (DPFC) |  |
|  | Brisch et al 2017 [90] (dorsal raphe) |  |

For further details of each study, see Tables 2-6 and Supplementary Tables 3 and 4.

^a^Using GFAP. ^b^Using glutamine synthetase.

CC: corpus callosum. ERC: entorhinal cortex. N.acc.: nucleus accumbens. OFC: orbitofrontal cortex. PT; planum temporale. TC: temporal cortex. WM: white matter. For other abbreviations, see Supplementary Table 5.
