# Supplementary material for: The neuropathology of bipolar disorder: systematic review and meta-analysis
Source: Mol Psychiatry. 2018 Aug 20;25(8):1787–808. doi: 10.1038/s41380-018-0213-3 (PMC6292507; doi:10.1038/s41380-018-0213-3)
Supplement: Supplementary file 2 — Supplementary Figures [file 41380_2018_213_MOESM2_ESM.pptx]

## Slide 1
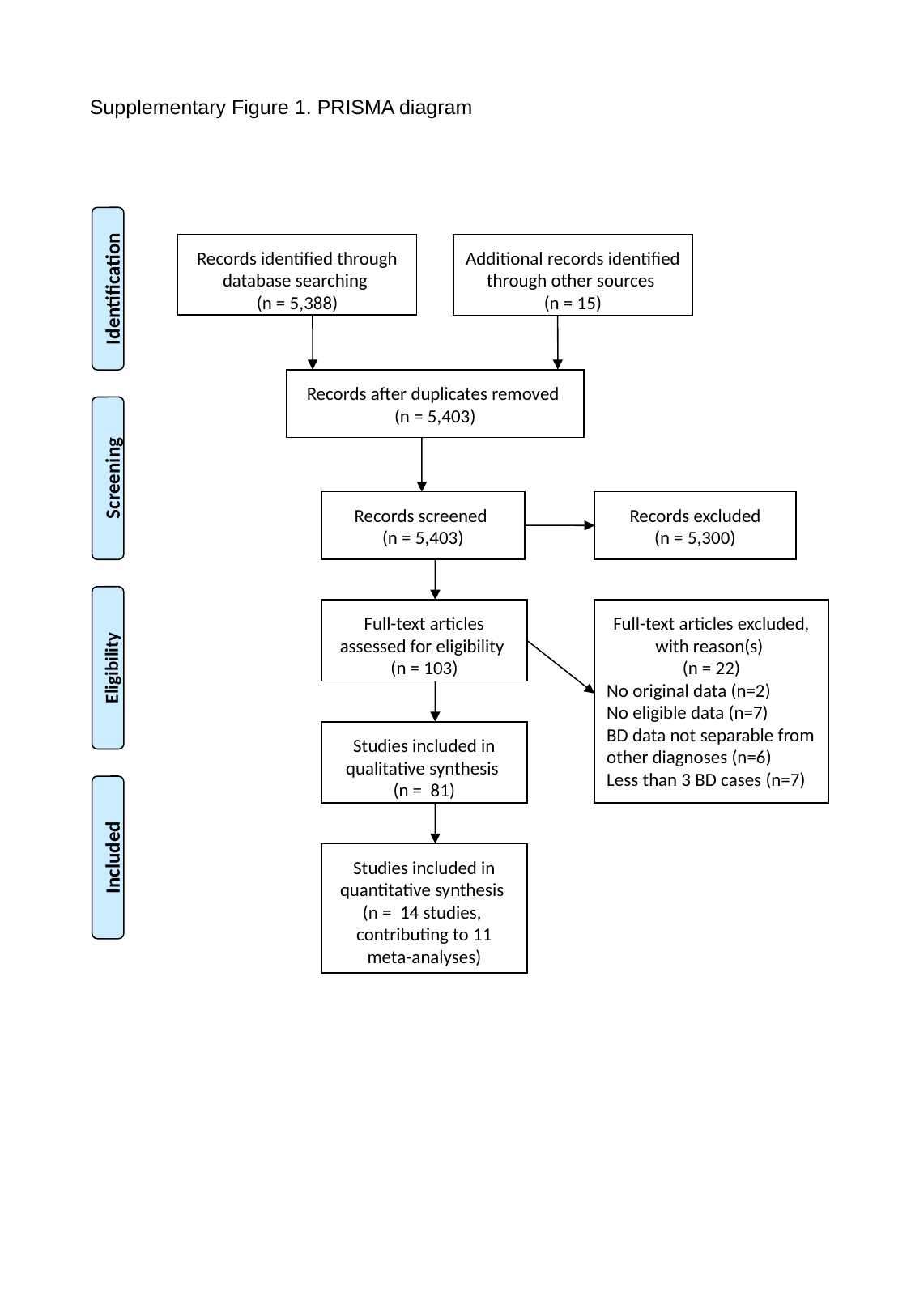

Records identified through database searching
(n = 5,388)
Additional records identified through other sources
(n = 15)
Identification
Records after duplicates removed
(n = 5,403)
Screening
Records screened
(n = 5,403)
Records excluded
(n = 5,300)
Full-text articles assessed for eligibility
(n = 103)
Full-text articles excluded, with reason(s)
(n = 22)
No original data (n=2)
No eligible data (n=7)
BD data not separable from other diagnoses (n=6)
Less than 3 BD cases (n=7)
Eligibility
Studies included in qualitative synthesis
(n = 81)
Included
Studies included in quantitative synthesis
(n = 14 studies, contributing to 11 meta-analyses)
Supplementary Figure 1. PRISMA diagram

## Slide 2
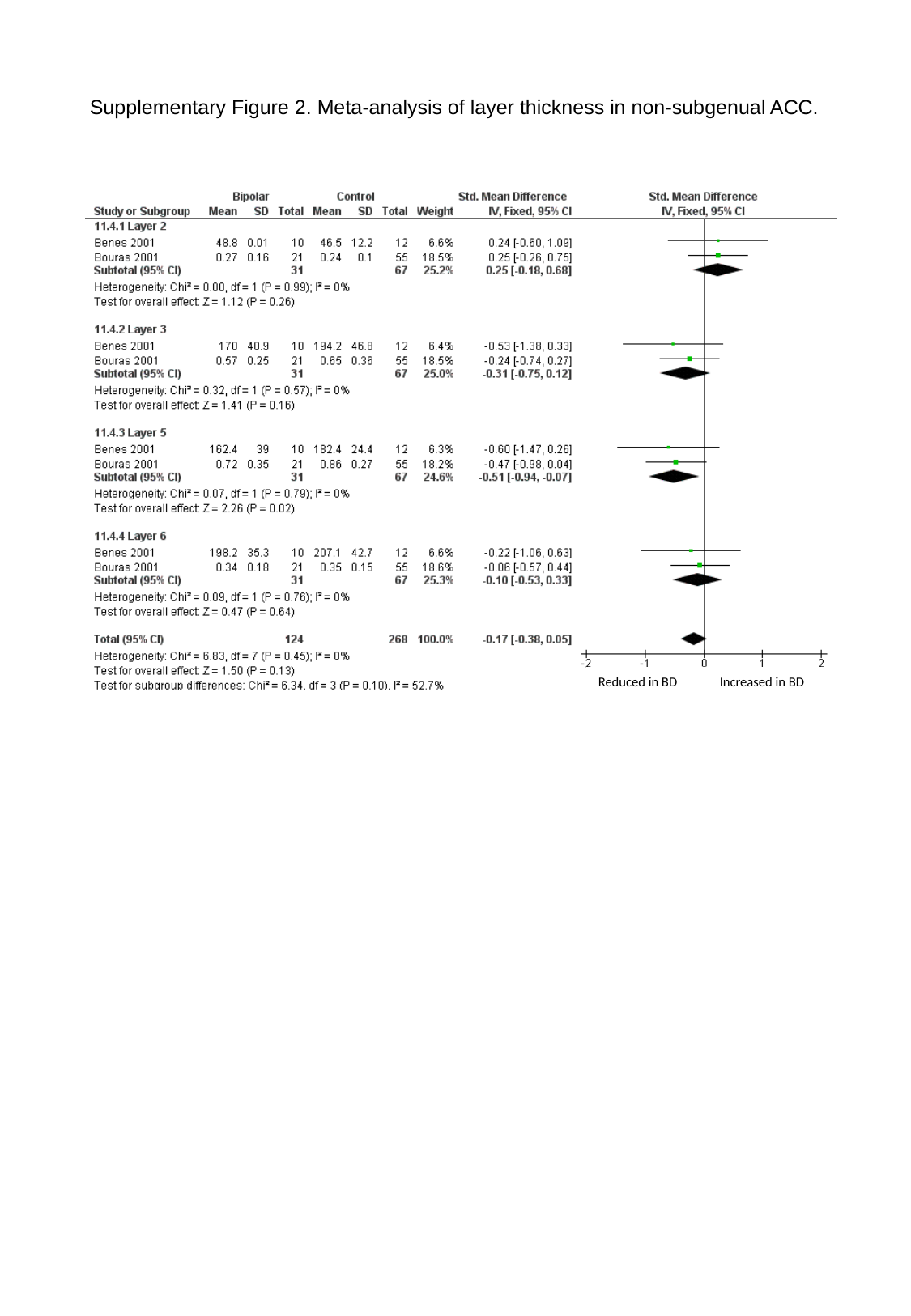

Supplementary Figure 2. Meta-analysis of layer thickness in non-subgenual ACC.
Reduced in BD	Increased in BD

## Slide 3
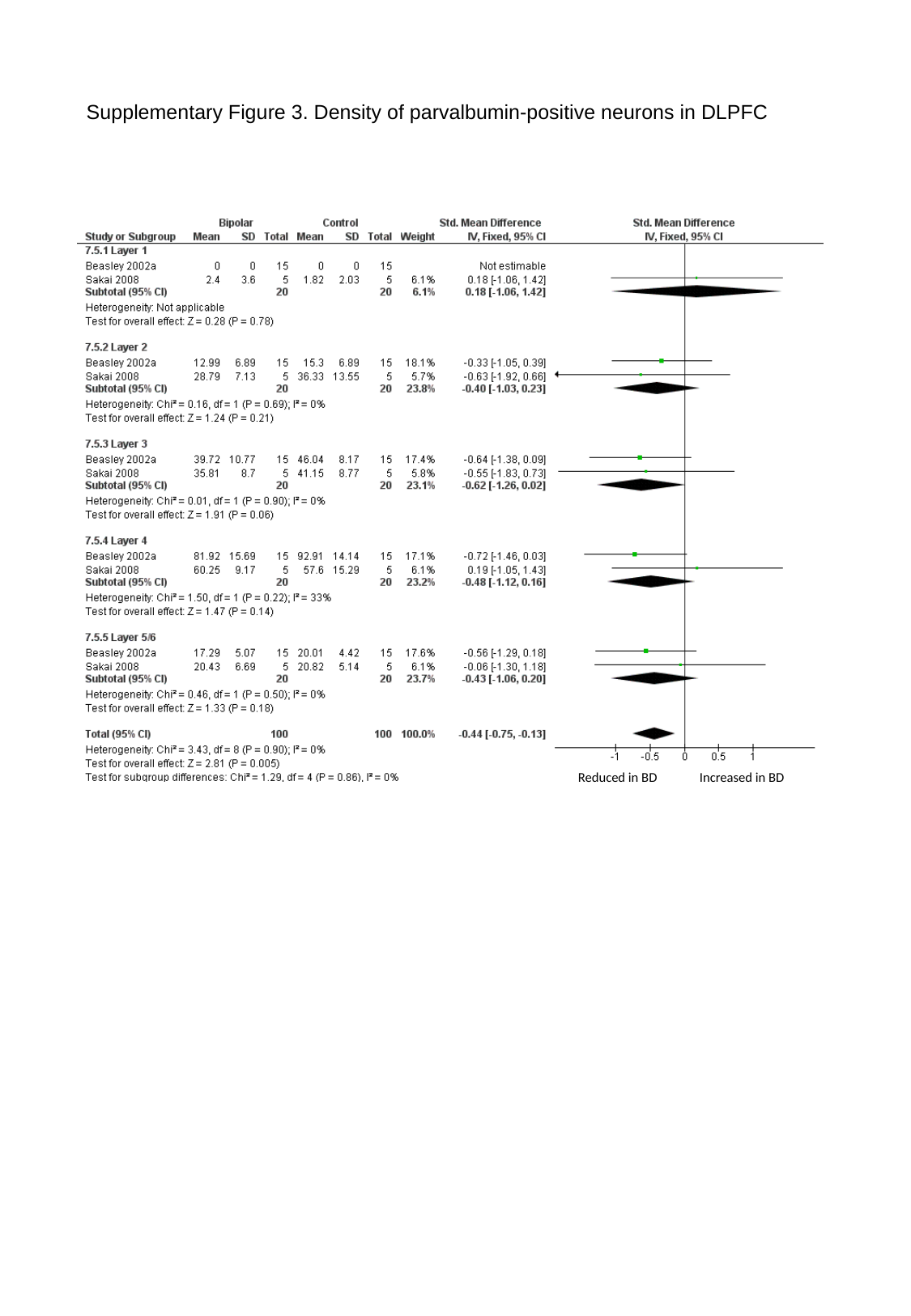

Supplementary Figure 3. Density of parvalbumin-positive neurons in DLPFC
Reduced in BD	Increased in BD

## Slide 4
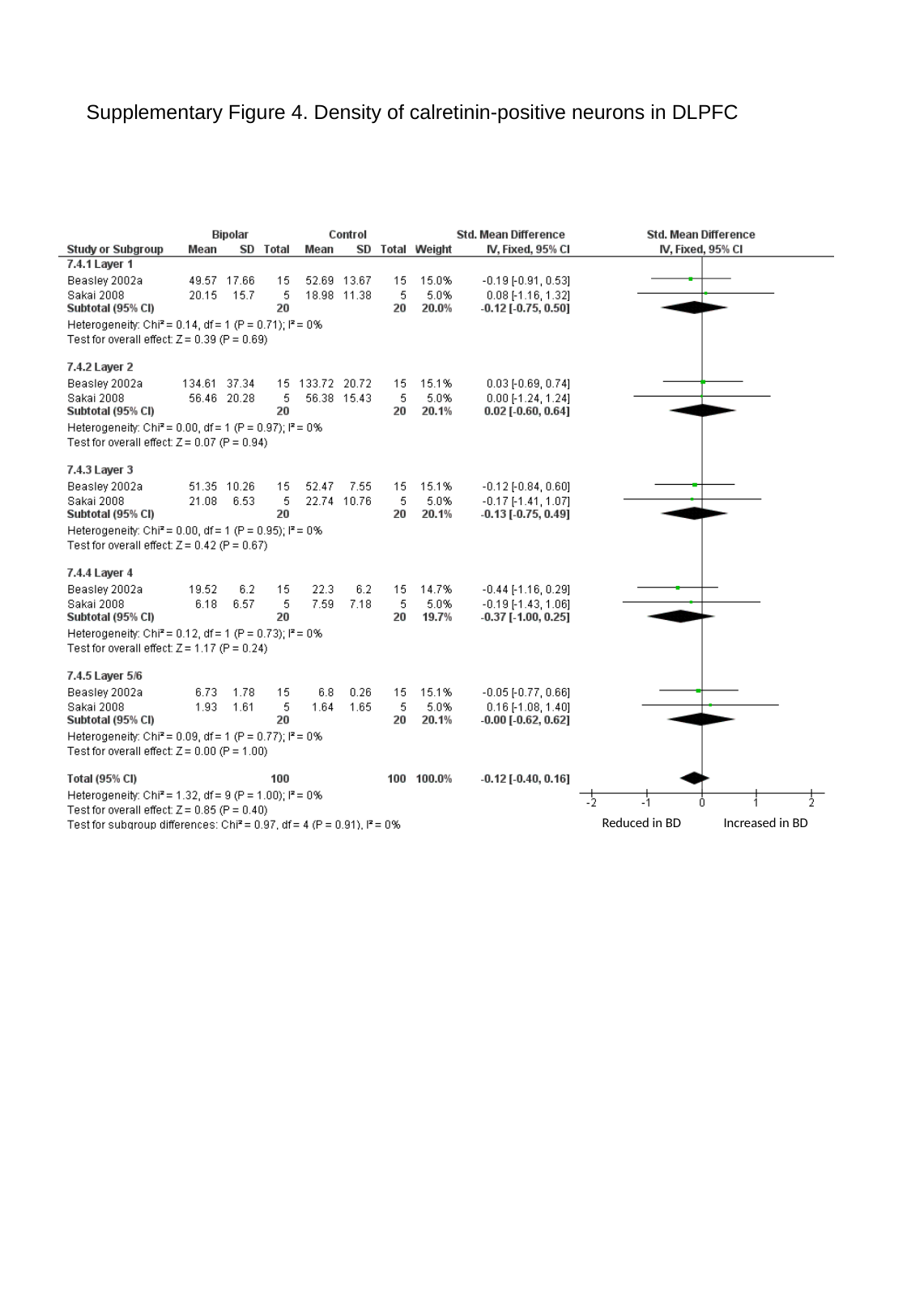

Supplementary Figure 4. Density of calretinin-positive neurons in DLPFC
Reduced in BD	Increased in BD

## Slide 5
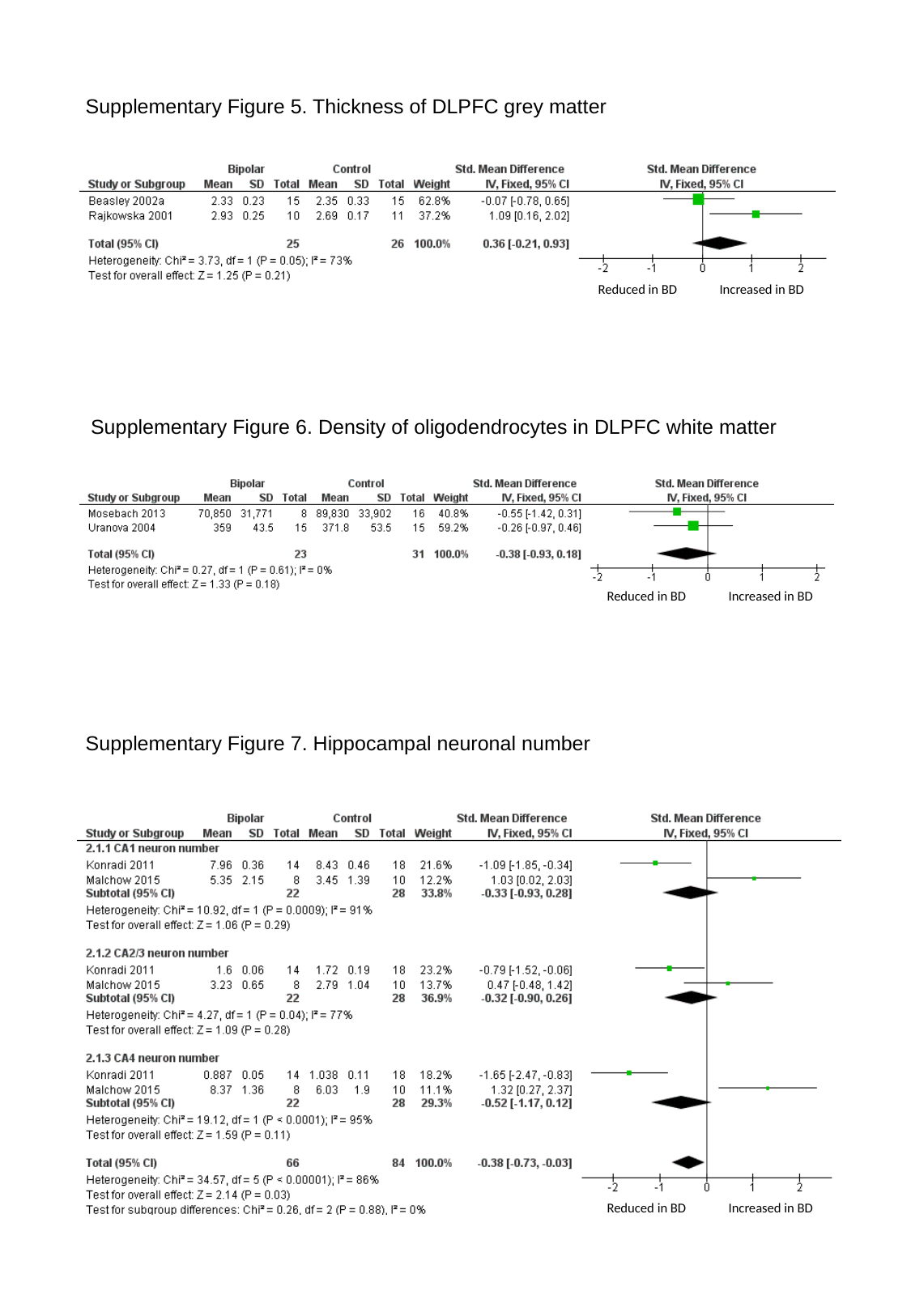

Supplementary Figure 5. Thickness of DLPFC grey matter
Reduced in BD	Increased in BD
Supplementary Figure 6. Density of oligodendrocytes in DLPFC white matter
Reduced in BD	Increased in BD
Supplementary Figure 7. Hippocampal neuronal number
Reduced in BD	Increased in BD
